# From Pollution to Value: Electrochemical Systems for Transforming Flue Gas into Chemicals and Fuels

**DOI:** 10.1002/adma.202509581

**Published:** 2025-09-02

**Authors:** Meng Wang, Adnan Ozden, Wang Tian, Jingyi Chen, Wei Chen, Lei Wang, Andrew Barnabas Wong, Yanwei Lum

**Affiliations:** ^1^ Department of Chemical and Biomolecular Engineering National University of Singapore Singapore 117585 Republic of Singapore; ^2^ Department of Mechanical and Nuclear Engineering Khalifa University Abu Dhabi 127788 UAE; ^3^ Center for Catalysis and Separations (CeCaS) Khalifa University Abu Dhabi 127788 UAE; ^4^ Department of Materials Science and Engineering National University of Singapore Singapore 117575 Republic of Singapore; ^5^ Department of Chemistry National University of Singapore 3 Science Drive 3 Singapore 117543 Republic of Singapore; ^6^ Department of Physics National University of Singapore 2 Science Drive 3 Singapore 117542 Republic of Singapore

**Keywords:** CCUS, CO_2_, electrocatalysis, electrochemistry, flue gas, sustainability

## Abstract

Electrochemical CO_2_ reduction reaction (CO_2_RR) can enable the production of fuels and chemicals from CO_2_ emissions. Direct flue gas conversion can address the emissions from hard‐to‐abate sectors, including cement, steel, and power generation industries. Such integration with SCO_2_RR can also decarbonize the petrochemical industry and eliminate separation and purification steps. Here, recent progress and emerging opportunities for transformative flue‐gas‐tolerant systems for producing fuels and chemicals are reviewed. The review begins by analyzing recent progress in direct flue gas conversion and highlighting emerging micro‐reaction environment features. Next, performance degradation mechanisms associated with flue gas conversion in CO_2_RR systems are discussed. Following this, the bicarbonate reduction reaction (BRR) and CO_2_RR from amine‐based capture as emerging strategies for high‐rate, selective, and stable electrosynthesis of chemicals from feeds containing low CO_2_ concentrations are highlighted. Finally, the mitigation of flue gas impurities such as oxygen, sulfur oxides, and nitrogen oxides, is discussed, and perspectives for emerging opportunities are shared.

## Graphical abstract

This review presents a comprehensive overview of the strategies whereby low‐concentration CO_2_ in flue gas can be directly valorized to value‐added chemicals and fuels. It covers recent progress in integrated CO_2_ capture and conversion, including amine and bi(carbonate) reduction‐based systems. This study also focuses on how the presence of impurities such as oxygen, sulfur oxides, and nitrogen oxides can be overcome.

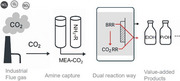

## 1 Introduction

The rising carbon dioxide (CO_2_) emissions require energy‐efficient, sustainable, and scalable CO_2_ utilization technologies.^[^ ^1^ ^]^ The electrochemical reduction of carbon dioxide (CO_2_RR) represents a critical strategy for mitigating anthropogenic emissions by converting CO_2_ into value‐added chemicals.^[^ ^2^ ^]^ Present‐day low‐temperature CO_2_RR achieves Faradaic efficiencies (FEs) of >80%,^[^ ^3^ ^]^ reaction rates of >200 mA cm^−2^,^[^ ^4^ ^]^ energy efficiencies (EEs) of >25%,^[^ ^5^ ^]^ and stable operation of >100 h.^[^ ^6^, ^7^ ^]^ Recent performance advances have motivated the shift in research priorities toward limiting challenges in terms of practicality and scalability,^[^ ^8^ ^]^ including CO_2_ separation and purification costs.

**Figure 1:**
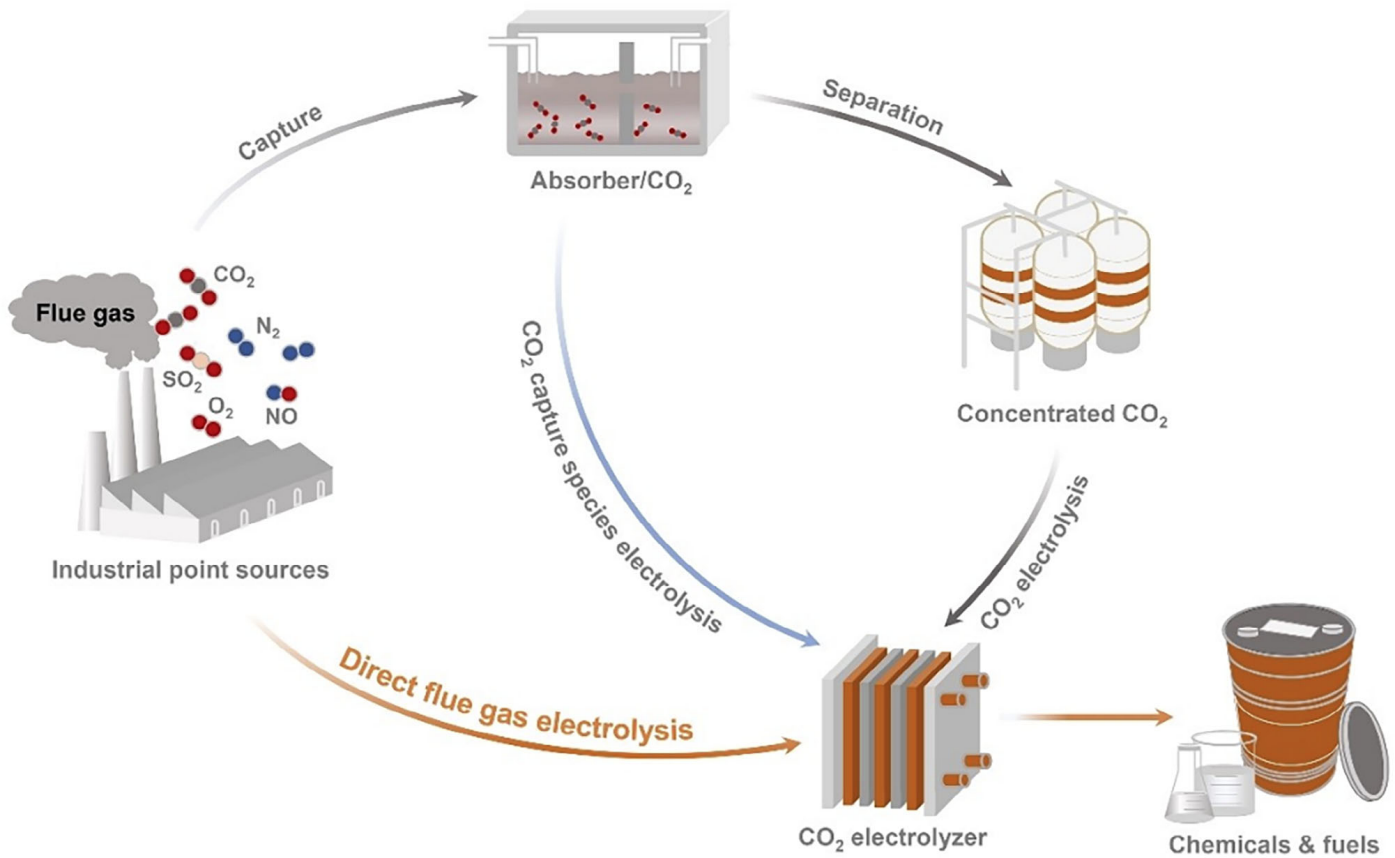
Pathways for flue‐gas utilization in the production of fuels and chemicals. Production of fuels and chemicals can be achieved via various pathways. Direct flue gas electrolysis is the pathway where fuels and chemicals are directly produced via the electrolysis of flue gas without prior capture, purification, and concentration. CO_2_ capture species electrolysis is the pathway where fuels and chemicals are produced via the electrolysis of CO_2_ capture liquids (i.e., BRR) upon the capture of CO_2_ from the flue gas stream. CO_2_ electrolysis is the pathway where fuels and chemicals are produced in a CO_2_ electrolyzer from concentrated CO_2_ (obtained via energy‐intensive capture, separation, and purification processes). Reproduced with permission.^[^ ^31^ ^]^ Copyright 2025, Wiley‐VCH.

However, today's laboratory‐scale research typically focuses on CO_2_RR from concentrated (i.e., >99.99%) CO_2_ feeds, which account for only 2.6% of global CO_2_ emissions.^[^ ^9^ ^]^ CO_2_ separation and purification processes are energy‐ and carbon‐intensive,^[^ ^10^ ^]^ typically necessitating heat sources from upstream steam or combustion of fossil fuels.^[^ ^11^ ^]^ Such processes also require high capital and maintenance costs. For example, monoethanolamine‐based CO_2_ purification requires sophisticated equipment, such as adsorption columns, heat exchangers, and stripping columns.^[^ ^12^, ^13^ ^]^ Overall, conventional capture and purification processes require US$90–140 tCO_2_^−1^.^[^ ^14^, ^15^ ^]^ Considering the multi‐carbon (C_2+_) nature of favorable CO_2_RR products and theoretical single‐pass conversion efficiencies (SPCE) limits in neutral‐ and alkaline‐media,^[^ ^16^, ^17^ ^]^ the CO_2_ separation and purification process could become prohibitive from a techno‐economic standpoint. For instance, the purification and separation could increase the production cost of ethylene and ethanol by up to 30%.^[^ ^18^, ^19^ ^]^ This economic burden has motivated efforts to utilize flue gas as an alternative feedstock for CO_2_ electrolyzers (**Figure** **1**). Flue gas is a predominant product of various hard‐to‐abate processes,^[^ ^20^ ^]^ including fossil‐fuel‐powered power plants, natural gas combined cycle plants, steel manufacturing, and cement production. Flue gas streams, depending on the source process, typically contain low‐concentration CO_2_ (≈1–30% vol.), carbon monoxide (CO) (5 ppm–80% vol.), nitrogen (N_2_) (5–80% vol.), water (H_2_O) (<5–18% vol.), and non‐negligible impurities, including oxygen (O_2_) (15–15% vol.), sulfur oxide (SO_2_) (10–1800 ppm), and nitrogen oxide (NO_x_) (50–1500 ppm) (**Table** **1**).^[^ ^21^ ^]^ Flue gas's compositional complexity introduces significant barriers to its direct utilization in CO_2_RR systems.

**Table 1:** Downstream composition of CO_2_‐emissions‐intensive processes.

Component	Coal‐fired power plant^[^ ^21^, ^47^ ^]^	Natural gas combined cycle^[^ ^21^, ^48^ ^]^	Steel manufacturing^[^ ^21^, ^40^ ^]^	Cement production^[^ ^21^, ^49^ ^]^
CO_2_	12–15%	3–5%	1–30%	11–14%
O_2_	3–4%	12–15%	<1%	2–14%
SO_2_	10–1800 ppm	<10 ppm	trace	100–1300 ppm
NO_x_	50–500 ppm	50 ppm	trace	100–1500 ppm
N_2_	75–80%	74–80%	5–47%	55–76%
H_2_O	5–14%	7–10%	<5%	7–18%
CO	<100–5000 ppm	<5 ppm	4–80%	160–1200 ppm

**Note**: The annual CO_2_ emissions from fuel combustion, steel manufacturing, and cement production are ≈34 GJ ton^−1^,^[^ ^50^ ^]^ ≈4 GJ ton^−1^,^[^ ^51^ ^]^ and ≈2 GJ ton^−1^,^[^ ^51^ ^]^ respectively. The concentrations are presented in vol%.

Two research focuses have emerged to address these barriers: electrocatalyst system design and impurity tolerance. In recent years, the field has started focusing on innovative catalyst and system design approaches to suppress the hydrogen evolution reaction (HER) by augmenting CO_2_ availability from low CO_2_ concentration feedstocks. In the context of CO_2_RR from dilute CO_2_, the research has concentrated on enhancing CO_2_ adsorption, tailoring surface wettability, and regulating local pH. There have also been rapid developments in alternative CO_2_ conversion routes, such as upgrading flue gas to chemicals and fuels via electrochemical reductions from bicarbonate solutions. This approach is referred to as the bicarbonate reduction reaction (BRR).

Another research focus is maintaining industrially relevant CO_2_RR performance in the presence of O_2_, SO_2_, and NO_x_. Various strategies have been developed to mitigate the detrimental effects of O_2_ impurities on CO_2_RR, leading to notable advancements in CO_2_RR from O_2_‐containing CO_2_ streams. The first strategy involves selective separation, such as coating the CO_2_RR catalyst with microporous polymers^[^ ^22^, ^23^ ^]^ to enhance CO_2_RR while removing O_2_ or utilizing liquid bicarbonate feedstocks to improve CO_2_RR through selective CO_2_ adsorption and O_2_ removal.^[^ ^24^ ^]^ The second strategy involves the chemical suppression of the oxygen reduction reaction (ORR) using bifunctional catalysts. The latter strategy focuses on lowering the energy barrier of CO_2_RR^[^ ^25^, ^26^ ^]^ and achieving O_2_‐tolerant CO_2_RR using acidic media.^[^ ^27^ ^]^ Likewise, there have also been efforts to design SO_2_‐tolerant catalysts and systems. These strategies involve modifying catalysts with ionomers and polymers to promote the transport of CO_2_ over that of SO_2_.^[^ ^28^ ^]^ The limited local availability of SO_2_ mitigates the SO_2_‐related challenges, including local pH reduction and active site blockage. Overall, by enabling direct utilization of flue‐gas streams, these strategies have opened the door to eliminating prohibitive upstream CO_2_ purification processes. A detailed analysis of recent progress and remaining challenges will motivate further breakthroughs.

Prior reviews focused on the advances and challenges associated with either dilute CO_2_ gas conversion via BRR^[^ ^29^, ^30^ ^]^ or impurity‐tolerant CO_2_RR catalysts/systems.^[^ ^31^ ^]^ For example, Wen et al.^[^ ^31^ ^]^ provided a focused review on catalyst and system design strategies for dilute and impurity‐containing CO_2_ upgrading, where BRR was not considered as a potential route for dilute CO_2_ upgrading. Likewise, other works overviewed the BRR and other electrochemical reactions/systems for dilute CO_2_ upgrading, where the scope was not extended to impurity‐tolerant catalyst and system designs.^[^ ^29^, ^30^ ^]^

Here, we provide a comprehensive overview of emerging catalyst, system, and process engineering approaches by simultaneously considering challenges associated with dilute CO_2_ upgrading and CO_2_ upgrading from impurity‐containing streams. We underscore impactful catalyst, system, and process design approaches to mitigate the challenges introduced from flue gas, including dilute and impurity‐tolerant flue gas upgrading. Furthermore, we suggest opportunities to achieve the remaining milestones toward large‐scale industrial adaptation of CO_2_RR technologies. The article contains five main sections, beginning with insights into flue gas electrolysis, proceeding with emerging flue‐gas conversion technologies, including CO_2_RR from dilute CO_2_ streams, BRR from dilute CO_2_ streams, CO_2_RR from amine‐based capture, and CO_2_RR from impurity‐containing flue gas streams. The article concludes by summarizing recent advances and the outlook for future scientific and technical advances. Overall, this review, by broadly considering the practical challenges associated with flue‐gas conversion, overviews the emerging flue‐gas conversion technologies and highlights their technological standing, strengths, and limitations.

## 2 Insights into Flue Gas Electrolysis

Direct upgrading of flue gas to valuable chemicals and fuels represents an appealing pathway, as it bypasses the capture and purification steps.^[^ ^32^, ^33^ ^]^ The composition of untreated flue gas shows variability depending on process specifications (Table 1). In general, the downstream concentration of CO_2_ is <30%, that of O_2_ is <15%, that of SO_2_ is <1800 ppm, and that of NO_x_ is <1500 ppm. The flue gas impurities can be classified as inert and active gases according to their physical and chemical properties.^[^ ^34^ ^]^ N_2_ is an inert gas that shows no participation in CO_2_RR. However, N_2_ can still reduce the partial pressure of CO_2_, and extreme lows could cause activity and selectivity loss.^[^ ^35^, ^36^ ^]^ The supply of CO_2_ at low concentrations to CO_2_RR systems could modulate micro‐reaction environment, CO_2_ coverage, intermediate adsorption and stabilization, and energy barriers in the formation of reactant intermediates. The modulation of the micro‐reaction environment causes substantial changes in product selectivity, reaction rate, energy efficiency, and operational stability. These changes typically originate from insufficient CO_2_ availability and suboptimal CO_2_ adsorption, shifting the reaction from C_2+_ products to C_1_ products. Computational and experimental studies show that low CO_2_ concentrations lower *CO_2_ coverage on copper (Cu) catalysts, reducing the coverage of *CO intermediate and promoting the protonation of *CO to *CHO—a key reaction intermediate toward methane.^[^ ^37^, ^38^ ^]^ Promoting CO_2_RR to C_2+_ products from dilute CO_2_ streams requires highly active and high‐surface‐area CO_2_RR catalysts.

Unlike inert gases, the active impurities, i.e., CO, O_2_, NO_x_, and SO_2_, exist in the flue gas with low compositions (Table 1). Thus, such active impurities typically possess a limited impact on the partial pressure of CO_2_. However, the reduction of some active species is thermodynamically more favorable than that of CO_2_.^[^ ^39^ ^]^ Thus, the active impurities typically undergo alternative, and thermodynamically more favorable reduction reactions (i.e., CO electroreduction reaction (CORR) or ORR) by utilizing electrons that would otherwise be utilized in CO_2_RR. For example, steel manufacturing yields downstream compositions containing 4%–80% CO.^[^ ^21^, ^40^ ^]^ Depending on the catalyst, CO can be either a product (i.e., on silver (Ag), gold (Au), and palladium (Pd))^[^ ^41^ ^]^ or an intermediate (i.e., on Cu‐ and Cu‐alloys)^[^ ^42^, ^43^ ^]^ on the way to C_2+_ products. CO_2_RR/CORR occur via distinct mechanisms and pathways. Thus, the presence of CO could change the product distribution and reaction kinetics. The introduction of CO in the reactant stream could shift the major electrolysis from ethylene to acetate by increasing *CO coverage and local pH.^[^ ^44^ ^]^ Additionally, increasing the partial pressure of CO in the CO_2_–CO stream promotes reaction rates.^[^ ^31^ ^]^ Despite these effects, the existence of CO in the reactant stream is not as problematic as SO_2_ and NO_x_ impurities. SO_2_ and NO_x_ impurities could be adsorbed on the catalyst surface under negative potentials.^[^ ^28^, ^45^ ^]^ They typically trigger surface reconstruction, deactivating the catalyst surface through active site blocking and domination of HER. Such physiochemical changes might be mitigated via periodic flushing of surface deposits.^[^ ^39^ ^]^ Additionally, SO_2_ could acidify the electrolyte and increase the temperature on the catalyst surface.^[^ ^46^ ^]^

Overall, direct flue gas conversion requires identifying effective strategies that could enable efficient, high‐rate, and stable CO_2_ upgrading from dilute CO_2_ streams as well as from those containing impurities (i.e., O_2_, SO_2_, and NO_x_). The following sections will provide a detailed overview of material‐ and system‐level strategies for performance and stability improvements in the electrosynthesis of chemicals and fuels from flue gas streams.

## 3 CO_2_RR from Dilute CO_2_ Streams

The low CO_2_ concentrations curtail the mass transport of CO_2_ over the active sites by favoring HER. Efforts have been dedicated to developing strategies that could improve the mass transport of CO_2_ to ensure CO_2_ availability at catalytically active sites or tune reaction pathways.^[^ ^27^, ^52^, ^53^ ^]^ Emerging control strategies include improving CO_2_ adsorption and capture, modulating reaction kinetics and intermediates, optimizing surface wettability (hydrophobicity and hydrophilicity), and regulating electrolyte pH. The following subsections provide an overview of emerging approaches that facilitate CO_2_RR from dilute CO_2_ streams.

### 3.1 Enhancing Local CO_2_ Availability and Adsorption

Various strategies have been explored to augment local CO_2_ availability by facilitating the adsorption of CO_2_ on the catalyst surface.^[^ ^54^, ^55^ ^]^

**Figure 2:**
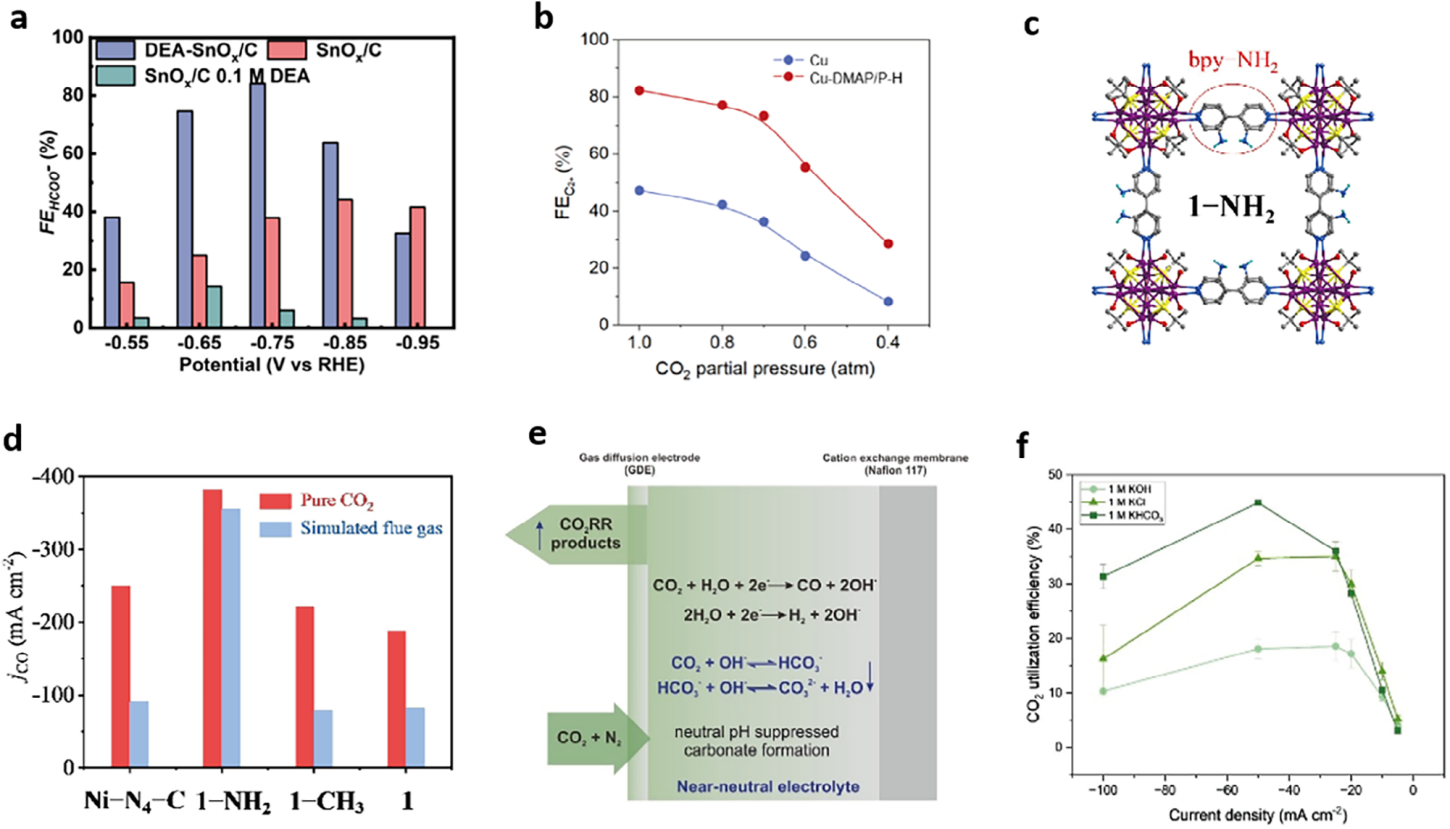
Micro‐reaction environment engineering strategies for efficient CO_2_RR from dilute CO_2_ streams. a) Formate FEs of various catalysts at various potentials in 0.5 m KHCO_3_ electrolyte from simulated flue gas. Reproduced with permission.^[^ ^59^ ^]^ Copyright 2021, ACS Publications. b) C_2+_ FEs of DMAP‐functionalized Cu catalyst (Cu‐DMAP/P‐H) and control Cu catalyst at various CO_2_ partial pressures at 200 mA cm^−2^. Reproduced with permission.^[^ ^60^ ^]^ Copyright 2024, Wiley‐VCH. c) Structure of Ag_12_bpy‐NH_2_ MOF catalyst for CO_2_RR‐to‐CO from dilute CO_2_ streams. Color codes: C, gray; Ag, violet; N, blue; O, red; S, yellow; H, cyan. d) CO partial current density (*j*_CO_) of Ag_12_bpy‐NH_2_ (1‐NH_2_), 1, Ag_12_bpy‐CH_3_ (1‐CH_3_), Ag_12_bpy (1‐NH_2_), and Ni‐N_4_‐C SACs from pure CO_2_ and simulated flue gas. c,d) Reproduced with permission.^[^ ^62^ ^]^ Copyright 2023, Wiley‐VCH. e) Schematic illustration of (bi)carbonate formation in neutral electrolyte. f) SPCE of CO_2_ in various electrolytes on NiCu catalyst. e,f) Reproduced with permission.^[^ ^82^ ^]^ Copyright 2025, Wiley‐VCH.

One effective approach involves modifying the catalyst surface with functional groups to augment CO_2_ availability.^[^ ^56^, ^57^, ^58^ ^]^ Cheng et al.^[^ ^59^ ^]^ demonstrated that functionalizing tin oxide (SnO_x_) catalyst with diethanolamine (DEA–SnO_x_/C) improves the interaction between the catalyst and CO_2_. The surface amino groups play multiple roles: enriching local CO_2_ concentration and facilitating key intermediate (OCHO^–*^) formation. As a result, the DEA–SnO_x_/C catalyst enables a formate FE of 84.2% and a formate partial current density (*j*_formate_) of 6.7 mA cm^−2^ at –0.75 V vs reversible hydrogen electrode (RHE) from simulated flue gas (15% CO_2_, 8% O_2_, balance with N_2_, vol.). In contrast, the unmodified SnO_x_/C catalyst exhibits a formate FE of <40% (**Figure** **2**a). Fu et al.^[^ ^60^ ^]^ reported that modifying the surface of Cu with 4‐dimethylaminopyridine (DMAP) enables the accumulation of electrons on the catalytically active sites of Cu catalyst, reducing the energy barrier for C–C coupling. As a result, DMAP‐functionalized Cu catalyst achieves considerably higher C_2+_ FEs compared to unmodified Cu catalyst under CO_2_ partial pressures from 0.4 to 1 atm at 200 mA cm^−2^ (Figure 2b). The improved C_2+_ productivity is ascribed to the enhanced availability and activation of CO_2_ enabled by the DMAP functionalization. Chen et al.^[^ ^61^ ^]^ ascribed the challenges associated with CO_2_RR from dilute feedstocks to limited mass transport, suboptimal thermodynamics, and sluggish kinetics. The authors sought to overcome these limitations via molecular tuning, which creates a micro‐reaction environment for successive CO_2_ capture and activation. This molecular‐tuning is performed by modifying cobalt phthalocyanine (CoPc) catalyst with poly(4‐vinypyridine) (P4VP). The P4VP‐modified CoPc catalyst achieves a CO FE of 90% and a CO partial current density (*j*_CO_) of 252 mA cm^−2^ from dilute CO_2_ (10% vol.) in an alkaline‐media membrane electrode assembly (MEA) electrolyzer. The CO productivity achieved with the P4VP‐modified CoPc catalyst is 2.24‐fold greater than that of the CoPc catalyst performing CO_2_RR under similar operating conditions. The enhanced CO_2_RR performance is ascribed to the multi‐functional roles of P4VP polymer in augmenting local CO_2_ availability and facilitating CO_2_ activation through electron donation to Co sites.

Efficient in situ CO_2_ capture and activation from dilute CO_2_ sources can also be achieved by increasing the catalyst's surface area via porous structures, such as metal–organic frameworks (MOFs). Liu et al.^[^ ^62^ ^]^ explored that a Ag cluster (Ag_12_)‐based MOF catalyst functionalized with aromatic amine groups (Ag_12_bpy‐NH_2_) exhibits significant performance improvements compared to un‐modified Ag catalysts. The enhanced CO_2_RR performance is ascribed to the improved CO_2_ capture enabled by the functionalized groups and the catalyst's porous structure (Figure 2c). When integrated into a MEA electrolyzer, the Ag_12_bpy‐NH_2_ catalyst achieves a CO FE of 96%, a current density of 120 mA cm^−2^, a full‐cell voltage of 2.3 V, and a full‐cell EE of 56%, and stable operation of 300 h from simulated flue gas (CO_2_:N_2_ = 15%/85% vol., at 298 K) (Figure 2d). These metrics exceed those of many benchmark electrolyzers performing CO_2_RR‐to‐CO from pure CO_2_ streams. Likewise, Zhao et al.^[^ ^63^ ^]^ implemented MOF modification on Bi catalyst (Bi‐HHTP, HHTP = 2,3,6,7,10,11‐hexahydroxytriphenylene) to achieve efficient CO_2_RR‐to‐formic acid from dilute CO_2_ (CO_2_/N_2_ = 15%:85%, vol.). The Bi‐HHTP catalyst enables a formic acid FE of 90% at a current density of 80 mA cm^−2^ and a full‐cell voltage of 2.6 V. At a constant cell voltage of 2.7 V (75–85 mA cm^−2^), the Bi‐HHTP catalyst enables continuous electrosynthesis of 200 mm pure formic acid solutions for 30 h. The Bi‐HHTP catalyst's advanced performance from dilute CO_2_ is ascribed to the enhanced CO_2_ capture enabled by microporosity and the lower Gibbs free energy of formation of *OCHO enabled by open Bi sites.

The prohibitive cost of a conventional adsorption‐based CO_2_ capture process has motivated innovative catalyst design approaches. Nabil et al.^[^ ^64^ ^]^ sought to achieve in situ CO_2_ regeneration and upgrading using a bifunctional gas diffusion electrode (BGDE) from a simulated flue gas containing 10% CO_2_ (vol.). The BDGE consists of a crystalline Cu catalyst and a high‐density polyethylene‐derived porous carbon physisorbent. By integrating CO_2_ separation and conversion, the BDGE creates a concentration gradient of non‐equilibrium binary gas flow (flue gas containing CO_2_ and N_2_), facilitating desorption of physisorbed CO_2_. Additionally, the use of high‐density polyethylene‐derived porous carbon as the physisorbent improves CO_2_ uptake. The combined effect of these two phenomena augments CO_2_ concentration in the micro‐reaction environment, enabling an ethylene FE of 45% at a current density of 80 mA cm^−2^.

### 3.2 Modulating Reaction Mechanisms and Intermediates

Modulating reaction kinetics and intermediates through catalyst design could provide a pathway to promoting CO_2_RR from dilute CO_2_. Wang et al.^[^ ^65^ ^]^ postulated that the CO_2_RR from dilute CO_2_ sources suffers from sluggish CO_2_RR kinetics. Accordingly, the authors implemented terephthalic acid (TPA) functionalization to improve the sluggish kinetics of CO_2_RR. The TPA functionalization strategy improves the CO_2_RR performance of various catalysts (Bi, Cu, and Zn) by modulating the binding energies of key intermediates, including *OCHO and *H. The TPA functionalized Bi catalyst achieves a twofold greater formate FE of 85.8% compared to its unmodified counterpart at 100 mA cm^−2^ from dilute CO_2_ (15% vol.). When integrated into an all‐solid‐state electrolyzer, the TPA functionalized Bi catalyst enables high‐purity formic acid production (50 mL of 0.49 m) for 30 h from dilute CO_2_ (5% vol.) at a current density of 100 mA cm^−2^. Xie et al.^[^ ^66^ ^]^ reasoned that the CO_2_RR from dilute CO_2_ feeds is curtailed by the surface adsorption strength of weak CO_2_RR intermediates and the suboptimal rate of CO_2_ conversion. The mechanistic studies indicated that under low CO_2_ concentrations, the RDS shifts from the *CO_2_ activation step to the formation of *COOH at the Cu^0^/Cu^1+^ interface. Accordingly, the authors sought to optimize the rate‐determining step (RDS) and reduce the thermodynamic adsorption limits of intermediates. By taking an interface boundary engineering approach, the authors sought to design a library of Cu catalysts with crystal structures abundant in Cu(111)/Cu_2_O(111) interface boundaries. The most promising catalyst exhibited a C_2+_ FE of 51.9 ± 2.8% and a C_2+_ partial current density (*j*_C2+_) of 34.5 ± 6.4 mA cm^−2^ from dilute CO_2_ (5% vol.). These findings agree well with those of prior studies proposing that the adjacent Cu^0^/Cu^1+^ interface serves as active sites to promote C_2+_ products (from pure CO_2_),^[^ ^67^, ^68^, ^69^ ^]^ and that the synergistic effects of Cu^0^ and Cu^1+^ active sites improve *CO dimerization and electron transfer.^[^ ^70^, ^71^, ^72^ ^]^

### 3.3 Tuning Surface Wettability

The surface hydrophobicity/hydrophilicity of catalysts plays a decisive role in CO_2_RR performance by controlling the reaction kinetics and mechanisms.^[^ ^73^, ^74^ ^]^ The wettability impacts the transport of CO_2_ and species and their underwater reaction dynamics. Optimal wettability can enable effective modulation of the adsorption/desorption of species in the electrical double layer.^[^ ^75^ ^]^ Particularly, at industrially relevant reaction rates, the mass transport profiles dominate CO_2_RR kinetics and pathways by modulating the surface coverage of CO_2_ and the binding energies of intermediates. Surface wettability could also control the H_2_O and CO_2_ ratio in the micro‐reaction environment.^[^ ^76^, ^77^ ^]^ Thus, such changes offer a pathway to optimize reaction kinetics and product selectivities. Polymer‐based surface modifications have been instrumental in modulating surface wettability. Chen et al.^[^ ^78^ ^]^ demonstrated that modifying Cu‐based GDE with a polythiophene (PT) polymer (featuring high porosity, high CO_2_ permeability, and low water uptake) optimizes the local H_2_O/CO_2_ ratio, enabling a C_2+_ FE of ≈75% at 500 mA cm^−2^ from a flue gas containing 50% CO_2_. Likewise, Kim et al.^[^ ^79^ ^]^ demonstrated that the surface H_2_O and CO_2_ concentrations are strongly correlated to CO_2_ partial pressure and catalyst surface properties. Multiphysics computational fluid dynamics (CFD) simulations suggest that water crossover in an MEA electrolyzer influences the competition between CO_2_RR and HER, enabling selective CO_2_RR‐to‐CO under low CO_2_ concentrations. Li et al.^[^ ^80^ ^]^ developed a bismuth‐poly (ionic liquid) (Bi‐PIL) composite catalyst in which the PIL backbone provides ionic conductivity and hydrophobicity, serving as a co‐catalyst and CO_2_ concentrator. The fine‐tuning of the hydrophobicity of the PIL backbone through crosslinking with biphenyl groups mitigates the flooding and solubility challenges concomitant with conventional ionic liquids (ILs). The Bi‐PIL composite catalyst exhibits a formate FE of 85% at cathodic potentials between –0.70–1.20 V vs reversible hydrogen electrode (RHE) (in a flow cell), from CO_2_ concentrations as low as 10% (vol.). The CO_2_RR‐to‐formate performance is largely ascribed to improved CO_2_ transport and enhanced CO_2_ absorption. When integrated into a solid‐state electrolyte (SSE) electrolyzer, the Bi‐PIL catalyst achieves production of 0.16 mol L^−1^ pure formic acid at 100 mA cm^−2^.

### 3.4 Tuning Electrolyte Identity and Concentration

The performance of CO_2_RR from dilute CO_2_ is highly influenced by electrolyte pH.^[^ ^81^ ^]^ Mahbub et al.^[^ ^82^ ^]^ reported that a near‐neutral electrolyte with buffering capacity enhances CO_2_RR at low CO_2_ concentrations (i.e., 2% vol.) over NiCu catalysts. In contrast, alkaline electrolytes cause excessive CO_2_ consumption because of the rapid reaction between CO_2_ molecules and OH^–^ ions. Milder electrolytes (i.e., 1 m KHCO_3_) mitigate (bi)carbonate formation compared to alkaline electrolytes (i.e., 1 m KOH), enabling higher local CO_2_ concentrations (Figure 2e). Augmenting local CO_2_ concentration enables higher SPCEs of 45% at 50 mA cm^−2^ from a simulated flue gas containing 2% CO_2_ (vol.) (Figure 2f). Acidic electrolytes minimize CO_2_ loss to (bi)carbonate formation. Wu et al.^[^ ^83^ ^]^ experimentally demonstrated that alkaline‐ and neutral‐media CO_2_RR systems fail to achieve high‐rate and efficient CO_2_RR from dilute CO_2_, due to the reactant CO_2_ loss to (bi)carbonate formation and triggering of HER. To promote CO_2_RR from dilute CO_2_ feeds (i.e., 5% vol.), the authors suppressed the reaction between locally generated OH^–^ ions and reactant CO_2_ by pursuing acidic‐media CO_2_RR. In acidic‐media CO_2_RR systems, balancing the influx of protons to the catalyst with the rate of electrochemically generated OH^–^ ions is critical to minimizing free protons, maintaining local alkalinity, and retaining regenerated CO_2_. These attributes are achieved by regulating proton transport via a solid‐state proton conductor. The micro‐reaction environment tuning strategy is effectively implemented on a hybrid catalyst comprising nanoporous Au and Ni single‐atom catalyst (SAC). The catalyst achieves a CO FE of 47.7% at 100 mA cm^−2^ from dilute CO_2_ (5% vol.). In contrast, under similar conditions (dilute CO_2_, 5% vol.), alkaline‐ and neutral‐media CO_2_RR systems deliver CO FEs of only 2.9% and 5.1%, respectively.

## 4 BRR from Dilute CO_2_ Streams

Despite material‐ and system‐level advances, most CO_2_RR systems still fail to combine feasibility enabling metrics (current density, energy efficiency, and stability) from dilute CO_2_, particularly in the presence of O_2_, SO_2_, and NO_x_. Researchers thus consider alternative strategies that can utilize low CO_2_ concentrations to produce CO_2_RR products with industrially relevant performance. To accomplish this, the BRR represents a promising technology that has seen recent rapid advances.^[^ ^84^, ^85^ ^]^ Unlike CO_2_RR, where gaseous CO_2_ is the reactant, BRR utilizes bicarbonate (HCO_3_^–^) ions as the reactant. The bicarbonate solutions can be chemically captured by purging dilute CO_2_ streams into alkaline solutions (i.e., KOH or NaOH) as follows:

(1) $$CO_{2\left(g\right)}+{\mathrm{OH}}_{\left(\mathrm{aq}\right)}^{-}\to {\mathrm{HCO}}_{3\left(\mathrm{aq}\right)}^{-}$$

Such alkaline solutions are available from other industrial processes, such as the chlor–alkali process—a key technology for present‐day direct air capture (DAC) processes. Thus, the bicarbonate solutions could be integrated as a downstream process of the existing DAC value chain, enhancing the economic benefit and sustainability of the carbon removal industry. The chemical sorption from alkaline solutions is typically more cost‐effective than purifying flue gas to concentrated CO_2_ streams.^[^ ^86^ ^]^ Further, the CO_2_RR from bicarbonate solutions could avoid problematic interactions with O_2_, owing to the low solubility of O_2_ in typical bicarbonate solutions.^[^ ^87^ ^]^ Here, this is particularly attractive because ORR is a key competing reaction that deteriorates the performance and stability of CO_2_RR in the presence of O_2_. SO_x_ from flue gas could react with water to form SO_3_^2–^ and SO_4_^2–^. However, according to Curtis's work,^[^ ^88^ ^]^ these two species have more negative reduction potential (–1.13 and –0.94 V vs SHE) than CO_2_RR to CO. Therefore, they claimed that BRR is intensive to the SO_x_ in flue gas input. Meanwhile, NO_2_^–^ and NO_3_^–^, generated through the adsorption of NO_x_ in alkaline solutions, have more positive reduction potentials than CO_2_. As a result, in their studies, NO_x_ impurities in flue gas can severely reduce the selectivity of the BRR process and should be removed before being purged into alkaline solutions. It should be noted that the influences of impurity gases in flue gas on BRR performances may be complicated and should be studied systematically in cell designs with different configurations and catalysts.

Another advantage of BRR lies in the wettability requirements from GDEs. In CO_2_RR systems, the catalytic reaction occurs at the three‐phase boundary (at the CO_2_/catalyst/electrolyte interface), necessitating a hydrophobic catalyst surface to ensure CO_2_ accessibility.^[^ ^89^ ^]^ In most cases, in direct CO_2_RR systems, the negative potentials cause electrowetting of carbon‐based GDEs,^[^ ^90^, ^91^ ^]^ leading to gradual electrolyte infiltration into the reactant/product carrying pathways. This phenomenon damages the stability of the triple‐phase boundary and triggers flooding. However, BRR requires no surface hydrophobicity for stable CO_2_RR. In contrast, in BRR, a hydrophilic interface is required because aqueous bicarbonate solution serves directly as the reactant. Thus, unlike CO_2_RR systems, BRR systems do not appear to suffer any performance decay because of hydrophobicity loss.

The following subsections provide an overview of the main BRR mechanisms, recent advances in dilute CO_2_ conversion to various products via BRR, and emerging electrolyzer design approaches for BRR.

### 4.1 Mechanisms of BRR

The field has reached a broad consensus on two primary reaction mechanisms. The first mechanism considers that HCO_3_^–^ ions react with protons to release CO_2_ on the catalyst surface as follows:

(2) $${\mathrm{HCO}}_{3\left(\mathrm{aq}\right)}^{-}+{\;H}_{\left(\mathrm{aq}\right)}^{+}\to CO_{2\left(g\right)}+H_{2}O_{\left(l\right)}$$

The in situ formed CO_2_ molecules then undergo CO_2_RR to produce valuable chemicals and fuels as follows:

(3) $$CO_{2\left(g\right)}+2H_{\left(\mathrm{aq}\right)}^{+}+2e^{-}\to CO_{\left(g\right)}+H_{2}O_{\left(l\right)}$$

**Figure 3:**
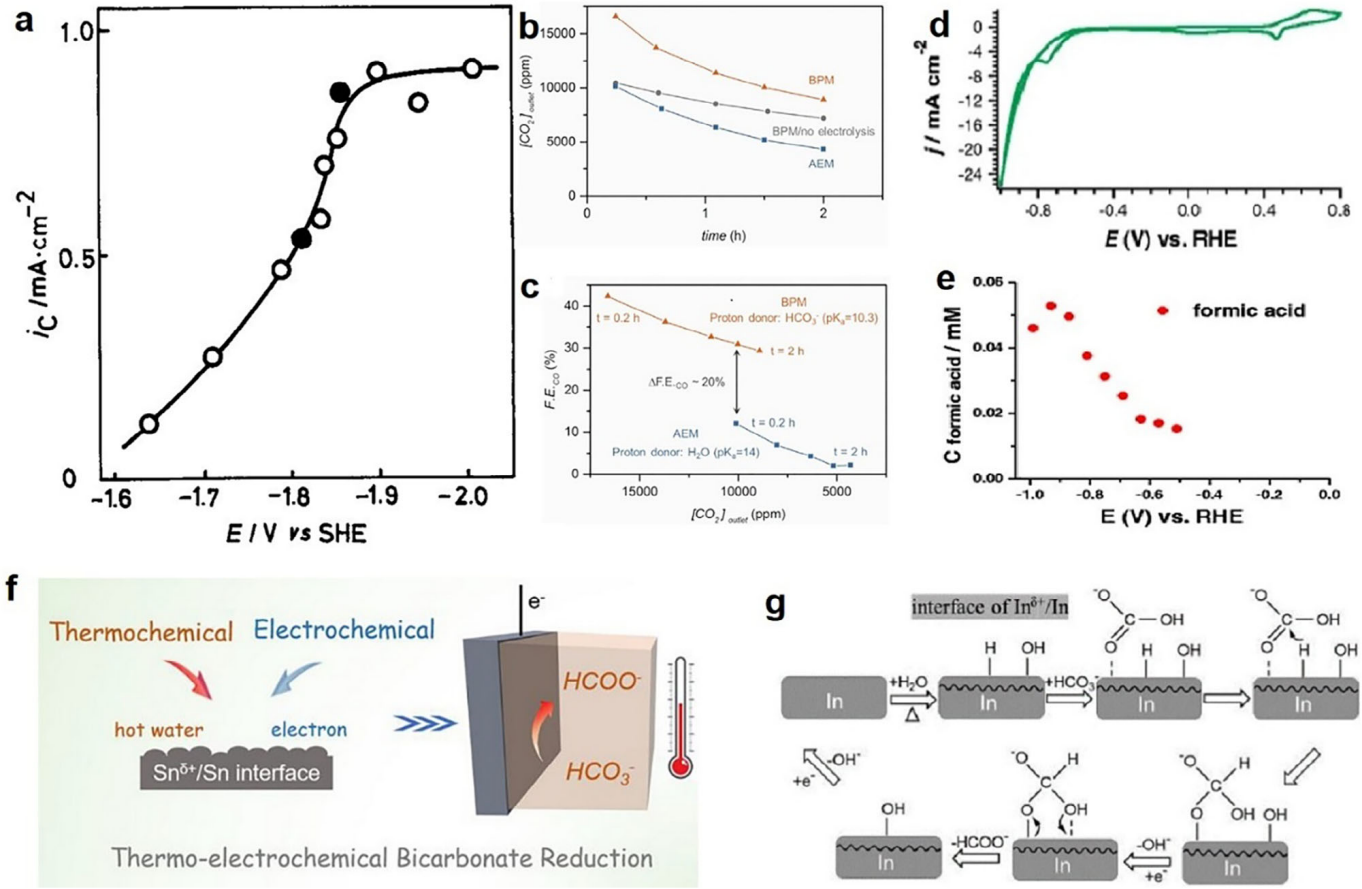
Insights into the mechanisms and performance of BRR. a) *j*_formate_ at various applied potentials with (open circle) and without (solid circle) N_2_ in electrolyte. Reproduced with permission.^[^ ^92^ ^]^ Copyright 1983, The Electrochemical Society. b) Temporal change in downstream CO_2_ concentration [CO_2_]_outlet_ during the electrolysis of 3.0 m KHCO_3_ at 100 mA cm^−2^ with a BPM (orange), an AEM (blue), or a BPM/no electrolysis (gray). c) CO FE as a function of [CO_2_]_outlet_ during 2‐h of electrolysis in 3 m KHCO_3_ solution at 100 mA cm^−2^ with a BPM (orange) and an AEM (blue). b,c) Reproduced with permission.^[^ ^93^ ^]^ Copyright 2019, Cell Press. d) CV of a polycrystalline Cu in 1.0 m KHCO_3_ at a scan rate of 20 mV s^−1^. e) Formic acid formation is detected with online HPLC using 1 m KHCO_3_ electrolyte without purging CO_2_. d,e) Reproduced with permission.^[^ ^97^ ^]^ Copyright 2013, Springer Nature. f) Schematic illustration of thermo‐electrochemical BRR on Sn catalyst. Reproduced with permission.^[^ ^94^ ^]^ Copyright 2023, American Chemical Society. g) Possible reaction process for BRR on In catalyst. Reproduced with permission.^[^ ^100^ ^]^ Copyright 2025, Chemical Society of Japan.

This mechanism is widely accepted and referred to as the “indirect route.” From our perspective, we would posit that the preference for the indirect route in the BRR field may potentially be attributed to the inherently difficult adsorption of bicarbonate ions on the catalyst surface based on structural and electronic factors. Structurally, HCO_3_^–^ is a non‐planar, asymmetric anion containing both a carboxylate group and a hydroxyl group. Electronically, its negative charge is more localized and tends to be strongly solvated through hydrogen bonding in aqueous solution, which hinders its ability to approach and interact directly with the catalyst surface. Furthermore, the lack of delocalized electronic structure hinders its capability to form stable coordination with translation metal sites. Therefore, the poor adsorption of bicarbonate on the catalyst surface hinders its direct electrochemical reduction. For example, in their seminal work,^[^ ^92^ ^]^ Hori and Suzuki explored BRR on a Hg catalyst. The system enables a *j*_formate_ of <1 mA cm^−2^ (**Figure** **3**a), which is two orders of magnitude smaller than the theoretical value if HCO_3_^–^ were directly reduced at the cathode. The *j*_formate_ shows no sensitivity to the convection of electrolyte, even at the limiting current range, indicating that aqueous HCO_3_^–^ does not serve as a reactive species in BRR. These findings suggest a mechanism where HCO_3_^–^ dissociates to reactive CO_2_ species prior to being upgraded to formate. Later, the crucial role of HCO_3_^–^‐derived CO_2_ in the electrochemical upgrading is further confirmed in a bipolar membrane (BPM)‐based flow cell.^[^ ^93^ ^]^ The downstream CO_2_ concentration ([CO_2_]_outlet_) with the BPM‐based flow cell is always greater than that measured with the anion exchange membrane (AEM) and BPM/no electrolysis (Figure 3b), confirming improved CO_2_ formation on the cathode of the BPM‐based system. Further, the linear relationship between CO FE and CO_2_ concentration for both BPM‐ and AEM‐based systems indicates that CO_2_ generated from the reaction between HCO_3_^–^ and proton is the active species during CO_2_RR (Figure 3c). Despite the widely accepted indirect route of bicarbonate, the possibility of other mechanisms has been considered,^[^ ^94^, ^95^, ^96^ ^]^ such as the one in which HCO_3_^–^ ions are directly reduced by catalysts as follows:

(4) $${\mathrm{HCO}}_{3\left(\mathrm{aq}\right)}^{-}+3H_{\left(\mathrm{aq}\right)}^{+}+2e^{-}\to CO_{\left(g\right)}+2H_{2}O_{\left(l\right)}$$

The direct reduction of HCO_3_^–^ has been proposed in the early stages of research on BRR. Several studies show that HCO_3_^–^ can be electrochemically reduced to formate on Pd catalysts, including supported Pd and bright Pd catalysts.^[^ ^97^ ^]^ Assessing liquid products by ^13^C nuclear magnetic resonance (NMR) spectroscopy, the electrochemical reduction of 13C‐enriched NaH^13^CO_3_ proves direct reduction of HCO_3_^–^ to formate on the Pd catalyst. Additionally, a characteristic cathodic peak is observed in cyclic voltammetry (CV) when (bi)carbonate electrolytes are used, which is absent in neutral and acidic media, suggesting a distinct electrochemical process of HCO_3_^–^ ions on the catalyst (Figure 3d).^[^ ^97^ ^]^ Further investigation into the potential dependence of formate yields on the applied potential (Figure 3e), at which formate production reaches its maximum, aligns with the peak current observed in the CV. This correlation suggests that the current peak can be attributed to the direct electrochemical reduction of HCO_3_^–^ to formate. The direct electrochemical reduction of bicarbonate is also reported in a homogeneous catalysis system.^[^ ^98^, ^99^ ^]^ Complex [Ru_III_(edta)(H_2_O)]^–^ combines with HCO_3_^–^ ions via water substitution to generate [Ru_III_(edta)(HCO_3_)],^2–^ which is then reduced by several steps to produce formate and regenerate [Ru_III_(edta)(H_2_O)]^–^. Recent findings on thermo‐electrocatalytic reduction have also challenged the long‐standing indirect mechanism for BRR, especially at elevated temperatures (Figure 3f). Pei et al.^[^ ^94^ ^]^ and Gu et al.^[^ ^100^ ^]^ explored the direct electroreduction of (bi)carbonate to formate using Sn and In catalysts under various conditions, including elevated temperatures (i.e., 100 °C) and high bicarbonate concentrations (i.e., 3 mol L^−1^ KHCO_3_). These studies propose a thermo‐electrocatalytic BRR mechanism, where heat and cathodic potentials synergistically promote the formation of dynamic metal/metal oxide (or hydroxide) interfaces (i.e., Sn^δ+^/Sn and In^3+^/In). These interfaces could facilitate the direct hydrogenation of bicarbonate (Figure 3g), bypassing dissolved CO_2_ as the intermediate. Additionally, the experiments based on pressurized and pure CO_2_ in non‐bicarbonate buffers yield much lower *j*_formate_, suggesting that HCO_3_^–^ concentration, not CO_2_, is the decisive factor in thermo‐electrocatalytic BRR. Although these findings do not invalidate the indirect mechanism under most conditions, they reveal the possibility of direct BRR or mixed direct and indirect mechanisms under specific electrochemical conditions.

### 4.2 Conversion of Dilute CO_2_ via BRR

Present‐day research on BRR targets various products in flow cells or MEA electrolyzers. Transition metal catalysts, including Ag, Au, Ni, and Co‐based catalysts, mainly generate CO as the dominant product, akin to the catalyst‐product relationship in CO_2_RR. Likewise, main group elements, including Sn and Bi, steer the reaction toward formate/formic acid.

#### 4.2.1 BRR to CO

Ag remains the most widely adopted catalyst for producing CO from BRR. Li et al.^[^ ^93^ ^]^ constructed a BPM‐based two‐electrode flow cell using Ag‐decorated porous carbon as a catalyst. Without a gaseous CO_2_ supply, the CO FE can still be as high as 81% at 25 mA cm^−2^ and 37% at 100 mA cm^−2^, comparable to the performance metrics of H‐type reactors based on CO_2_‐saturated electrolyte. To increase the loading of Ag on GDE, Lees et al.^[^ ^101^ ^]^ adopted physical vapor deposition (PVD) and spray deposition method, reaching a high CO FE of 82% ± 2% at 100 mA cm^−2^. In addition to Ag nanoparticles, Ag foam is also used as a free‐standing catalyst in BRR systems. Ag foam presents several advantages: fabrication and scaling convenience, high hydrophilicity, high mechanical strength and chemical durability, and high tolerance to common BRR impurities (i.e., NO_3_^–^ and SO_4_^–^). Zhang et al.^[^ ^102^ ^]^ reported a bicarbonate electrolyzer based on Ag foam. The system shows a CO FE of 59%, whereas the control system based on Ag/carbon composite electrodes exhibits a lower CO FE of 33%. The structural stability of Ni foam improves the operational stability compared to the control system based on Ag/carbon composite electrode. The Ni‐foam‐based electrolyzer shows a 3% performance loss during 80 h of continuous BRR, whereas the control system demonstrates a greater performance loss of 16%. At 4 atm, a CO FE of 95% is achieved at 100 mA cm^−2^. The improved performance under pressurized conditions is enabled by the mechanical integrity of Ni foam. Burgers et al.^[^ ^103^ ^]^ attributed the performance degradation to the trace amount of metal impurities in the electrolyte. The authors sought to overcome the impurity‐related performance loss by using ethylenediaminetetraacetic acid (EDTA), which forms complexes with metal impurities. As a result, the system shows a minor performance degradation of 4% after 6 hours of BRR. Fink et al.^[^ ^104^ ^]^ also used Ag foam as a catalyst in a BPM flow cell. The system yields a CO FE of 30% in saturated LiHCO_3_ solution. However, a higher CO FE of ≈80% is obtained with electrolytes containing larger cations (i.e., Cs^+^). The cation identity can facilitate BRR by tuning the interfacial electric field in the micro‐reaction environment. Beyond Ag‐based catalysts, Ni‐ and Co‐based SACs are also reported as promising catalysts for BRR.^[^ ^105^, ^106^, ^107^, ^108^ ^]^ Kong et al.^[^ ^105^ ^]^ developed Ni–N–S catalysts by introducing S into the second shell of the Ni center, which increases the electron density in the vicinity of the N atoms. As a result, the adsorption of *H on the N atom is stabilized, facilitating the proton‐coupled electron transfer (PCET) process. Lawson et al.^[^ ^107^ ^]^ prepared a soluble CoPc derivative (cobalt phthalocyanine molecule with four positively charged trimethylammonium groups), in which the decorated functional groups improve water solubility and stabilize catalytic intermediates through a high‐density positive charge.

#### 4.2.2 BRR to Formate

In the early stages of BRR research, a purified Hg electrode was adopted as the catalyst.^[^ ^92^ ^]^ As a main group element, Hg produced formate as the dominant product. Gutiérrez‐Sánchez et al.^[^ ^109^ ^]^ reported a novel BRR improvement approach that inhibits the proton donor ability of HCO_3_^–^. The approach involves modifying the surface of SnO_2_ with cationic surfactants, such as hexadecyltrimethylammonium chloride (CKC). The design limits the transport of polar water and HCO_3_^–^ ions to the catalyst surface while allowing for the diffusion of non‐polar CO_2_ molecules. The catalyst upgrades CO_2_ to formate while suppressing HER. The BRR system achieves a formate FE of 70% in 2 m bicarbonate solution. Nomoto et al.^[^ ^110^ ^]^ developed a bicarbonate electrolyzer that employs a porous membrane between the hydrophilic Bi cathode and the PEM. The system optimizes in situ CO_2_ generation and regulates pH gradient. The electrolyzer achieves formate FEs of 91.2% and 84.6% at 100 mA cm^−2^ and 300 mA cm^−2^, respectively. Hu et al.^[^ ^111^ ^]^ synthesized In‐Bi alloy catalysts with various In loadings (0–15 wt%) via hydrothermal and sequential electroreduction. The pure Bi nanoparticles show a formate FE of ≈70% at 60–100 mA cm^−2^. After alloying with In, a notable performance improvement is observed, such that the formate FE increases from ≈70% to ≈83.5% at 60 mA cm^−2^. The inclusion of In suppresses the HER, evidencing its HER inhibition effect. At the early stage of BRR research, the low bicarbonate conversion was a concern, especially when compared to the relatively adequate CO_2_ conversion achieved in direct CO_2_RR. Fortunately, with years of development, bicarbonate conversion has been greatly improved, now catching up with and even surpassing the CO_2_ conversion achieved in current CO_2_RR systems. For example, Li et al.^[^ ^112^ ^]^ directly sprayed commercial Sn nanoparticles and multi‐wall carbon nanotubes mixture onto hydrophilic carbon paper. Combined with a near‐neutral‐pH cation exchange membrane (CEM) and a glass fiber intermediate layer, the electrolyzer exhibited an impressive bicarbonate‐to‐formate conversion up to 96%, which can be explained by the buffered pH at the cathode and suppressed HER.

Besides Sn‐ and Bi‐based catalysts, Ru‐complexes also hold promise for formate production via BRR.^[^ ^98^ ^]^ A few notable works showcase the feasibility of homogeneous catalysis for BRR.^[^ ^96^, ^98^, ^113^ ^]^ Specifically, [Ru_III_(edta)(H_2_O)]^–^ combines with HCO_3_^–^ via water substitution to generate [Ru_III_(edta)(HCO_3_)],^2–^ which is then reduced by two electrons to generate unstable [Ru_I_(edta)(HCO_3_)]^4–^ species. The process proceeds with the generation of formate and regeneration of [Ru_III_(edta)(H_2_O)]^–^ via PCET. The process eliminates the need for in situ CO_2_ generation. The high formate FE and the absence of CO or H_2_ as by‐products further support the BRR. A subsequent review by the same research group elaborates on the electrochemical, thermochemical, and photocatalytic applications of this Ru(III) system,^[^ ^96^ ^]^ highlighting its versatility and distinguishing it from surface‐bound heterogeneous catalysts.

#### 4.2.3 BRR to Other Hydrocarbons

In addition to CO and formate, other hydrocarbons could be produced via BRR. Lees et al.^[^ ^114^ ^]^ fabricated a BPM‐based bicarbonate electrolyzer based on a porous Cu catalyst. BPM provides protons to the catalyst, converting KHCO_3_ to *i*‐CO_2_, which is then reduced to methane. Additionally, by adding a cationic surfactant to the electrolyte, the HER can be suppressed, further promoting methane formation. The reactor achieves a methane yield of 34% at 120 mA cm^−2^, which is higher than the previous maximum of 3%.^[^ ^115^ ^]^ Lee et al.^[^ ^116^ ^]^ developed a BRR system that converts bicarbonate to C_2+_ products via an innovative Cu/Ag double‐layer catalyst. The Ag layer first reduces *i*‐CO_2_ to CO, and then the Cu layer produces C_2+_ products under high CO concentration. The optimization of the bilayer configuration, metal composition (Cu/Ag), dual ion exchange membrane (Sustainion/Nafion), and local hydrophobicity (i.e., polytetrafluoroethylene (PTFE)) creates a highly alkaline micro‐reaction environment with high water content. Such an environment is favorable for the generation of C_2+_ products and suppression of HER.^[^ ^117^ ^]^ Ultimately, a C_2+_ FE of 41.6 + 0.39% is achieved at 100 mA cm^−2^.

### 4.3 Electrolyzer Design for BRR

The early stage BRR research focused on testing Hg catalysts in the simplest and most convenient setup, a three‐electrode cell.^[^ ^92^ ^]^ However, the suboptimal proton flux, product crossover, and high system resistance limit the industrial prospect of such systems. The field has started transferring the learnings on catalyst design and micro‐reaction environment tuning to scalable MEA electrolyzers. In such systems, an ion‐conducting membrane is sandwiched between a porous anode and a cathode, and flow‐field plates are used to feed electrolytes to the anode and cathode. Due to the compact configuration, the gap between active layers can be minimized, lowering cell potential. In CO_2_RR systems, cathode and anode catalysts can be physically and electronically separated by an AEM,^[^ ^118^ ^]^ a CEM,^[^ ^119^ ^]^ or a BPM.^[^ ^120^ ^]^ However, in BRR systems, sufficient H^+^ flux is required to react with bicarbonate to form *i*‐CO_2_, which is then reduced through a PCET process.

**Figure 4:**
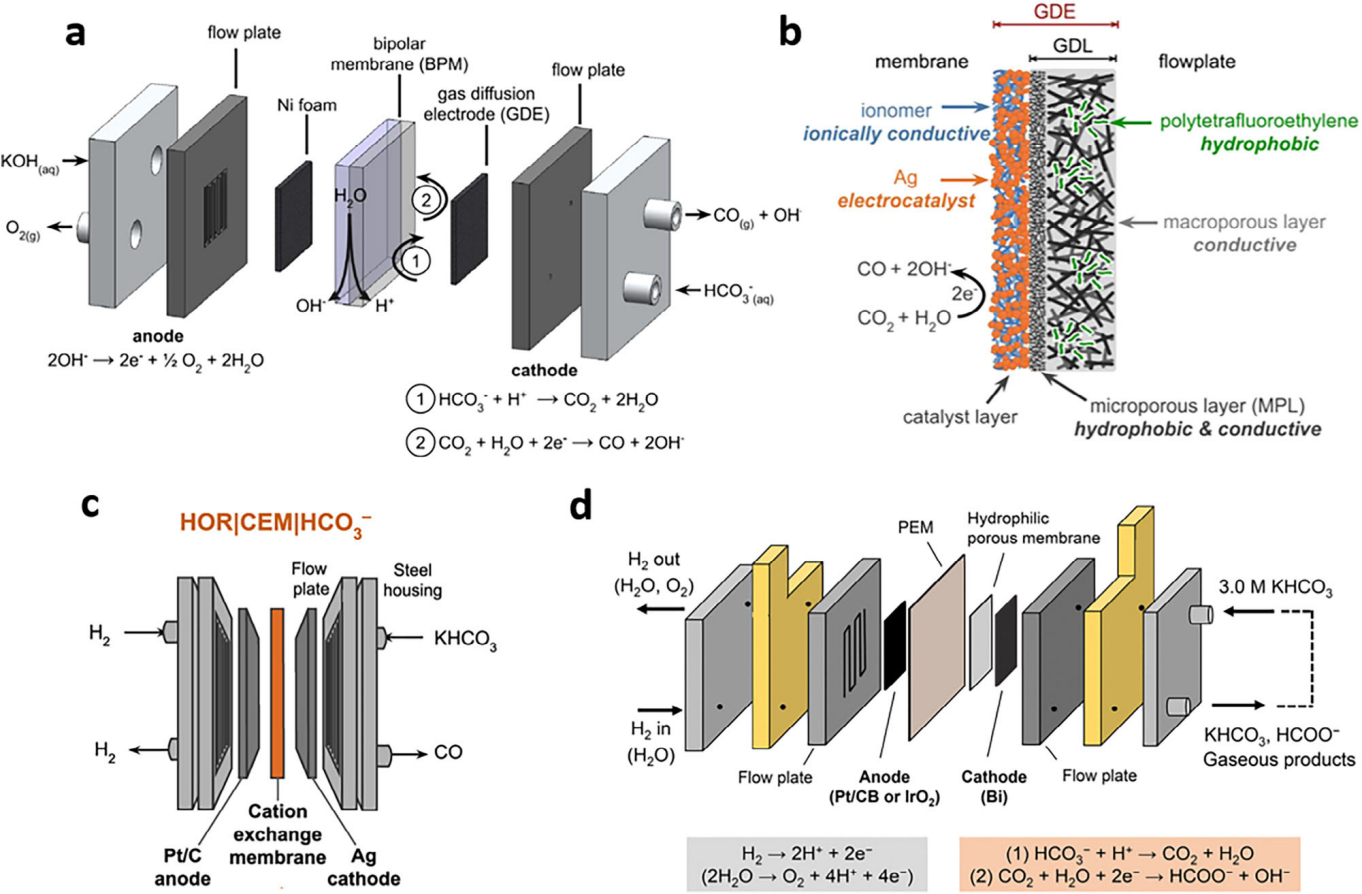
Reactor design considerations for flue‐gas upgrading to various products via BRR. a) Schematic illustration of a BPM‐based electrolyzer for BRR. b) Schematic illustration of a GDE along with its constituents, i.e., CL, MPL, and GDL. a,b) Reproduced with permission.^[^ ^101^ ^]^ Copyright 2020, American Chemical Society. c) Schematic illustration of a CEM‐based electrolyzer performing HOR at the anode (using Pt/C catalyst) and BRR at the cathode (using Ag catalyst). Reproduced with permission.^[^ ^123^ ^]^ Copyright 2022, American Chemical Society. d) Schematic illustration of a BRR electrolyzer with a hydrophilic porous membrane in‐between the PEM and the cathode catalyst. Reproduced with permission.^[^ ^110^ ^]^ Copyright 2024, Royal Society of Chemistry.

Early BRR research focused on BPM‐based electrolyzers to convert bicarbonate to electrochemically active CO_2_ through continuous H^+^ supply (**Figure** **4**a), creating a regime with CO_2_ concentration exceeding the CO_2_ solubility limit. Accordingly, the CO FE can be improved by 20% compared to that of AEM electrolyzers.^[^ ^93^ ^]^ Further, the proton flux from BPM also inhibits the pH increase in the catholyte at large current densities, mitigating the formation of electrochemically inert species (i.e., CO_3_^2–^). Unlike the gaseous CO_2_ supply in CO_2_RR, in situ CO_2_ is generated by the reaction between H^+^ and bicarbonate, which are all from electrolytes. A hydrophilic interface benefits wettability and transport of HCO_3_^–^ ions. Thus, the design considerations for BRR systems show differences compared to those for CO_2_ electrolyzers.^[^ ^101^ ^]^ For instance, a GDE without a microporous layer (MPL) and a hydrophobic agent (PTFE) shows an impressive CO FE of 82% (Figure 4b), indicating that the elimination of hydrophobic components (i.e., polymeric binder (Nafion) and PTFE) has no adverse effect on performance. This trend is opposite to the performance‐ and stability‐improving roles of hydrophobic components in CO_2_RR systems.^[^ ^121^ ^]^ Likewise, in another study,^[^ ^109^ ^]^ BRR adopting a cation exchange ionomer (i.e., Nafion) as the binder exhibits a lower formate FE than that adopting an anion exchange ionomer (i.e., Sustainion): 27% vs 25% formate FE at 25 mA cm^−2^. This trend might be attributed to the fact that the hydrophilic backbone in the anion exchange layer vs the hydrophobic backbone in the cation exchange layer provides hydrophilicity, forming a more favorable micro‐reaction environment for BRR. Similar performance trends are observed when main group elements, including Bi, are utilized to form the catalyst layer (CL). More specifically, the formate FE in the BPM‐based electrolyzer is 35% higher than that in the AEM‐based MEA electrolyzer.^[^ ^122^ ^]^

**Figure 5:**
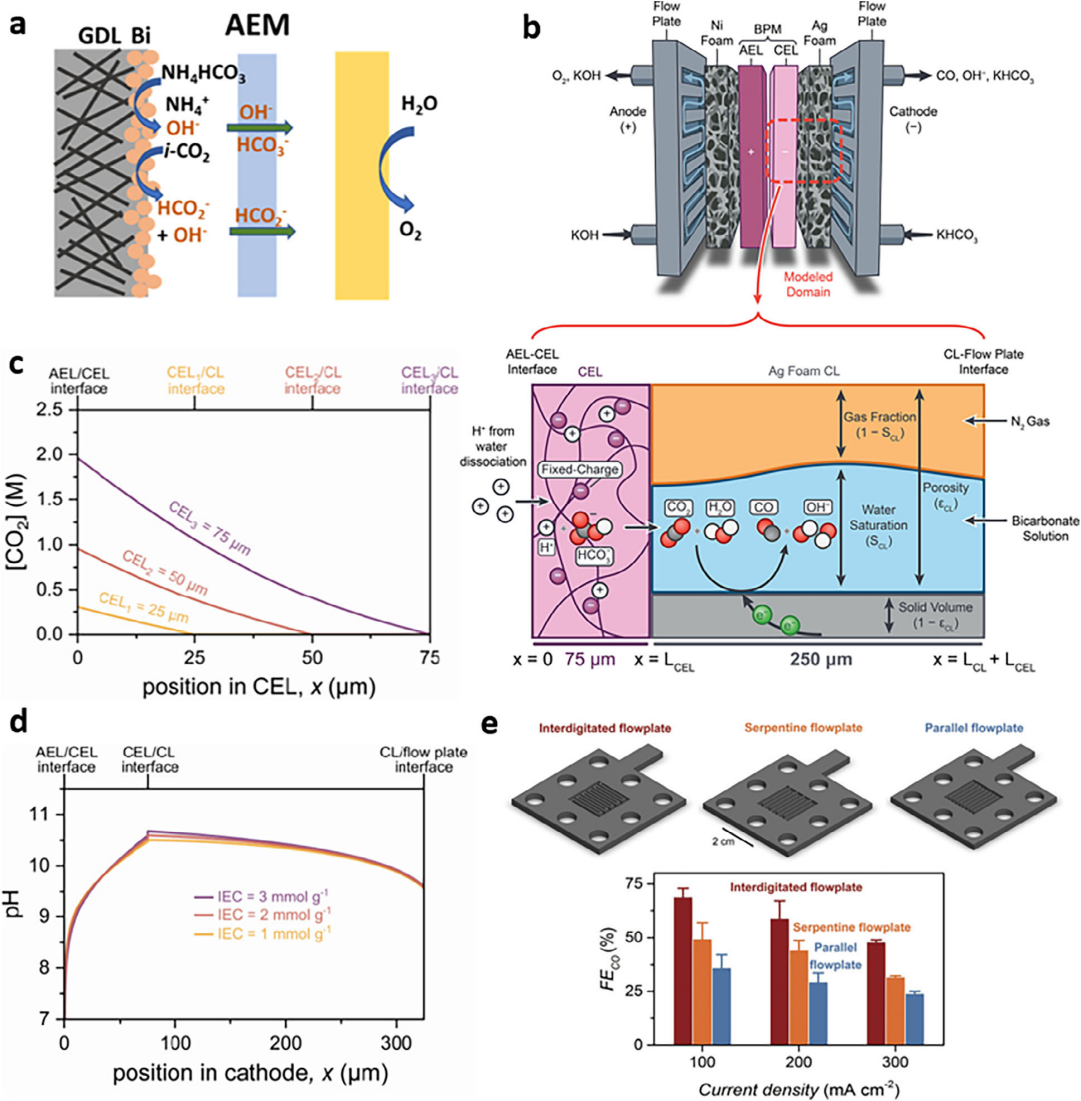
Reactor design considerations for flue‐gas upgrading to various products via BRR. a) Schematic illustration of the cathodic micro‐reaction environment of electrolyzers that are equipped with AEMs and fed with NH_4_HCO_3_.^[^ ^125^ ^]^ b) Schematic illustration of a bicarbonate electrolyzer comprised of flow‐field plates, a Ni foam anode, a BPM, and an Ag foam cathode. The continuum model describes the CEL, CL, and three‐phase boundary (interface of gas, liquid, and solid). Boundary conditions are defined at the AEL/CEL interface (x = 0), CEL/CL interface (x = L_CEL_), and at the CL/flow‐field plate interface (x = L_CEL_+L_CL_). c) Correlation between the IEC and pH of the micro‐reaction environment. d) Correlation between the CEL thickness and CO_2_ concentration. b–d) Reproduced with permission.^[^ ^126^ ^]^ Copyright 2022, American Chemical Society. e) Integrated, serpentine, and parallel cathodic flow‐field plate patterns and corresponding CO FEs at various current densities. Reproduced with permission.^[^ ^102^ ^]^ Copyright 2022, Royal Society of Chemistry.

Nevertheless, BPMs typically suffer high cell voltages due to the water dissociation overpotential between the anion and cation exchange layers. The high voltages motivate researchers to consider other membrane types, such as CEMs.^[^ ^108^, ^110^, ^123^, ^124^ ^]^ CEMs provide proton flux to the catholyte to generate *i*‐CO_2_ for the next reduction process. In CEM‐based electrolyzers, the anodic oxygen evolution reaction (OER) can be replaced with the hydrogen oxidation reaction (HOR) to achieve voltage reductions (Figure 4c). Due to the lower thermodynamic potential of HOR (0 V vs. RHE) than that of OER, the electrolyzer achieves a *j*_CO_ of 220 mA cm^−2^ and a CO_2_ SPCE of 40% at a cell voltage of only 2.3 V. In contrast, the BPM‐based electrolyzer requires a cell voltage of 12.7 V at 200 mA cm^−2^. The continuous proton flux to the micro‐reaction environment enables sufficient *i*‐CO_2_. However, excessive proton concentrations trigger HER, reducing the FE toward CO or formate. Therefore, a buffer solution^[^ ^123^ ^]^ or porous layer^[^ ^110^ ^]^ can be introduced in‐between the CEM and the cathode catalyst to avoid excessive acidity. Specifically, introducing a layer of porous and hydrophilic cellulose esters between the PEM and the cathode (Figure 4d) increases the formate FE from 47.3% to 90%. Overall, a moderate local pH—rather than excessively low or high pHs—is ideal for selective BRR. However, in terms of cell voltage and energy consumption, AEMs are more advantageous than CEMs and BPMs. Liu and coworkers developed an ammonia‐mediated CO_2_ capture and ammonium bicarbonate (NH_4_HCO_3_) electrolyzer, where AEM is used as the ion conductor (**Figure** **5**a).^[^ ^125^ ^]^ Owing to the favorable thermal deposition of NH_4_HCO_3_ at 40 °C, *i*‐CO_2_ is generated by giving OH^–^, achieving three‐times higher *i*‐CO_2_ concentration and a higher formate FE compared to what can be achieved in a bicarbonate electrolyzer. Moreover, the adoption of AEM reduces cell voltage by ≈35%, improving energy efficiency.

Electrochemical BRR involves the diffusion of HCO_3_^–^ ions and H^+^ at the CEM/catalyst interface to generate *i*‐CO_2_. Therefore, the mass transfer of different species influences the performance of BRR. Lees et al.^[^ ^126^ ^]^ developed a model to explore the mass transfer processes in the CL and cation exchange layer (CEL) of a bicarbonate electrolyzer (Figure 5b). The model attributed the lower CO FEs at high current densities to the sluggish CO_2_ mass transport within the CL. The model predicted the steady‐state concentration of CO_2_ to be less than 1 mm throughout the CL. Such a low CO_2_ concentration originates from rapid *i*‐CO_2_ consumption. Modulating the material properties at the CEL and CL interface improves CO_2_ transport. However, a CEL composed of an ionomer possessing high ion exchange capacity (IEC) could amplify the electric field, enhancing charge repulsion at the AEL/CEL interface. More specifically, an ionomer loading of 3 mmol g^−1^ inhibits the OH^–^ transport at the CL/CEL interface (Figure 5c), increasing CO FE. Thicker CELs extend the proton diffusion path from the AEL/CEL interface to the CEL/CL interface. A shorter pathway decreases the acidity in the CL, limiting the proton transport and suppressing HER. Additionally, a thinner CL with higher porosity could improve *i*‐CO_2_ generation (Figure 5d) by shortening the bicarbonate diffusion path from the flow plate through the CL to the CEL/CL interface. Overall, from the mass transfer standpoint, a thick CEL with a high‐IEC ionomer and a thin and porous CL improves the BRR performance. Such a configuration could also establish an acidic membrane layer and an alkaline CL, promoting *i*‐CO_2_ generation and suppressing HER.

Process parameters, such as pressure, flow‐field geometry, flow rate, and temperature, also impact the mass transfer and thermodynamics of BRR.^[^ ^102^, ^127^ ^]^ High electrolyzer pressure is beneficial for BRR, because it can increase the solubility of CO_2_ in the electrolyte, limiting the migration of *i*‐CO_2_ in the form of gas bubbles.^[^ ^102^ ^]^ The flow‐field geometry (i.e., interdigitated, serpentine, and parallel) also impacts the BRR performance (Figure 5e). The discontinuity of interdigitated flow‐field plates forces the electrolyte to pass through the electrode to reach the outlet channel, causing higher convective mass transfer compared to serpentine and parallel flow‐field plates. Among all the tested configurations, the electrolyzer adopting an interdigitated flow‐field plate shows the highest CO FE of 69% ± 4% at 100 mA cm^−2^. The flow rate could influence product selectivity and outlet concentrations in different ways. On one hand, fast flow rates could shorten the residence time of in situ generated gas bubbles (CO and H_2_), which could reduce active sites by covering the catalyst surface. Additionally, high flow rates could create turbulence within the cell, promoting convective mass transfer of species. As a result, a 25% higher formate FE is achieved when the flow rate is increased from 0.5 to 5 mL min^−1^. However, a low flow rate prolongs the catholyte residence time, increasing downstream formate concentration and decreasing recovery cost.^[^ ^127^ ^]^ Likewise, reaction temperature influences BRR. Zhang et al.^[^ ^102^ ^]^ postulated that high temperatures (20 °C vs 70 °C) could increase *i*‐CO_2_ generation by accelerating bicarbonate dissociation kinetics, causing a higher CO FE (78% vs 59%) and a lower cell voltage (3.5 V vs 3.6 V). Higher temperatures could also improve ionic conductivity, which could reduce cell voltage by decreasing the resistance. Nevertheless, higher temperatures could also decrease CO_2_ solubility and increase bicarbonate solubility, reducing *i*‐CO_2_ and FE. Overall, the influence of temperature on BRR depends on experimental conditions, warranting comprehensive evaluation and case‐specific modulation.^[^ ^102^, ^127^ ^]^ The cation identity also influences the product distribution of BRR. Fink et al.^[^ ^104^ ^]^ explored the effect of alkali cations (Li^+^, Na^+^, K^+^, and Cs^+^) on the product distribution of BRR. Results showed that the CO FE increases with rising cation radius (from Li^+^ to Cs^+^), attributable to the modulation of interfacial electric field and increasing charge density.

To conclude, electrochemical BRR, as a promising route to utilize low‐concentration CO_2_ sources, offers unique advantages. BRR is compatible with existing DAC technologies. The BRR systems also offer intrinsic advantages, such as high O_2_ tolerance and very impressive bicarbonate conversion up to 96% when integrated with DAC technologies. Such systems also show no sensitivity to flooding. Despite their intrinsic advantages, the BRR technology encounters several critical challenges. The BRR systems, compared to direct CO_2_RR systems, suffer relatively poor selectivity, attributable to limited understanding of reaction mechanisms and fundamental phenomena. Additionally, the strong proton donation ability of carbonate and the favorable hydrophilic interface favor competing HER. As a newly explored reaction, the limited mechanistic understanding and the high propensity toward competitive HER result in the lower FE of BRR compared to CO_2_RR. The heavy dependence on BPMs for BRR is another challenge, warranting low‐overpotential and stable alternatives. The literature has a dearth of systematic studies on process integration that encompasses the capture of low concentration CO_2_ to generate bicarbonate solutions, BRR kinetics, mechanisms, and performance, and the pH swing process for electrolyte recirculation. Lastly, an understanding of the electrode/electrolyte interface can profoundly influence the kinetics and thermodynamics of BRR and CO_2_RR.

Based on these insights, the techno‐economic feasibility of direct CO_2_RR and BRR routes can be evaluated from the following key perspectives. When optimized, reports on BRR can achieve higher carbon and CO_2_RR utilization efficiency than CO_2_RR because it avoids carbon loss as carbonate. Unlike CO_2_RR systems with AEM, where CO_2_ reacts with hydroxide and forms (bi)carbonate that crosses to the anode, BRR systems utilize BPMs to generate CO_2_ locally at the cathode, preventing ion crossover and thereby retaining more carbon within the system. On the other hand, the utilization of BPM increases the cell voltage of BRR due to the intrinsic 0.83 V thermodynamic requirement for water dissociation, as well as due to additional overpotential from the water dissociation catalyst. Due to the inherently high ohmic drop and water dissociation potential of BPM in BRR, their current densities are generally lower than those of typical CO_2_RR, especially under conditions of high BRR selectivity. However, by adopting a CEM, the current density in BRR can be increased to 1000 mA cm^−2^, comparable to that achieved in state‐of‐the‐art CO_2_RR systems. By using bicarbonate directly as the feedstock, BRR avoids CO_2_ emissions associated with carbonate‐to‐CO_2_ conversion in the calciner, which is a necessary step in CO_2_RR. As a result, BRR systems can achieve negative CO_2_ emissions across all cell voltages when the FE exceeds 20%.

## 5 CO_2_RR from Amine‐Based Capture

**Figure 6:**
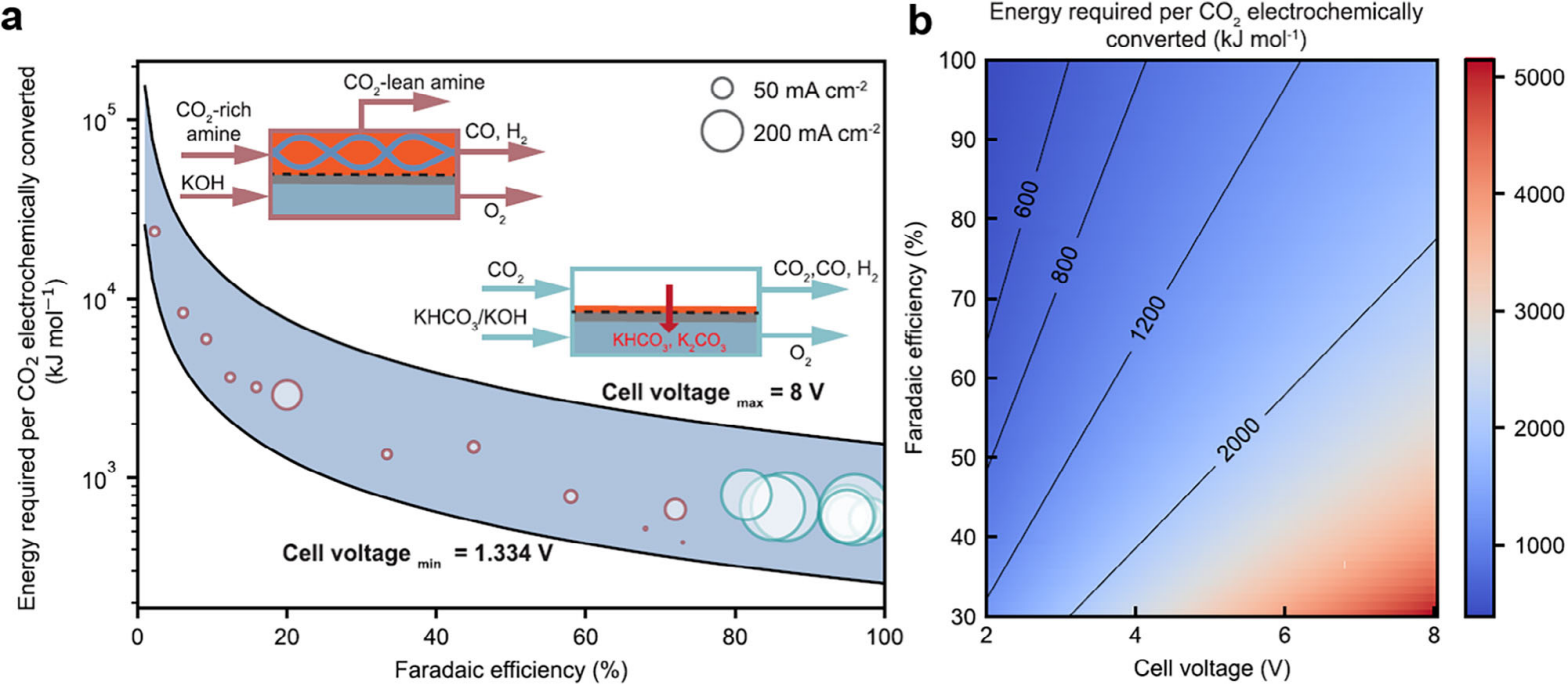
Energy input of CO electrosynthesis via CO_2_RR from amine‐based capture solutions. a) A comparison of the energy consumption, CO faradaic efficiency, and cell potentials over integrated and gas‐fed CO_2_RR. b) The role of CO faradaic efficiency and cell voltage in determining the overall energy consumption of CO_2_RR to produce CO. Reproduced with permission.^[^ ^135^ ^]^ Copyright 2022, Springer Nature.

CO_2_RR from amine‐based capture solutions is another emerging approach for chemical electrosynthesis from dilute CO_2_ streams. The approach involves integrating CO_2_ capture and electrochemical conversion to improve the overall energy efficiency. The process is also compatible with the catalyst and electrolyzer design approaches developed for other dilute CO_2_ upgrading approaches. Recent findings have shown that the catalysts that are selective for gas‐fed CORR are also efficient for catalysis of CO_2_RR in the amine capture media.^[^ ^128^, ^129^, ^130^, ^131^ ^]^ Traditionally, CO_2_ captured in amine‐based solvents, such as monoethanolamine (MEA), requires thermal desorption before further electrochemical reduction, which adds significant energy and operational cost.^[^ ^132^, ^133^, ^134^ ^]^ **Figure** **6**a,b compares the energy performance of these two electrolysers to convert CO_2_ to CO,^[^ ^135^ ^]^ revealing the extra energy cost.

**Figure 7:**
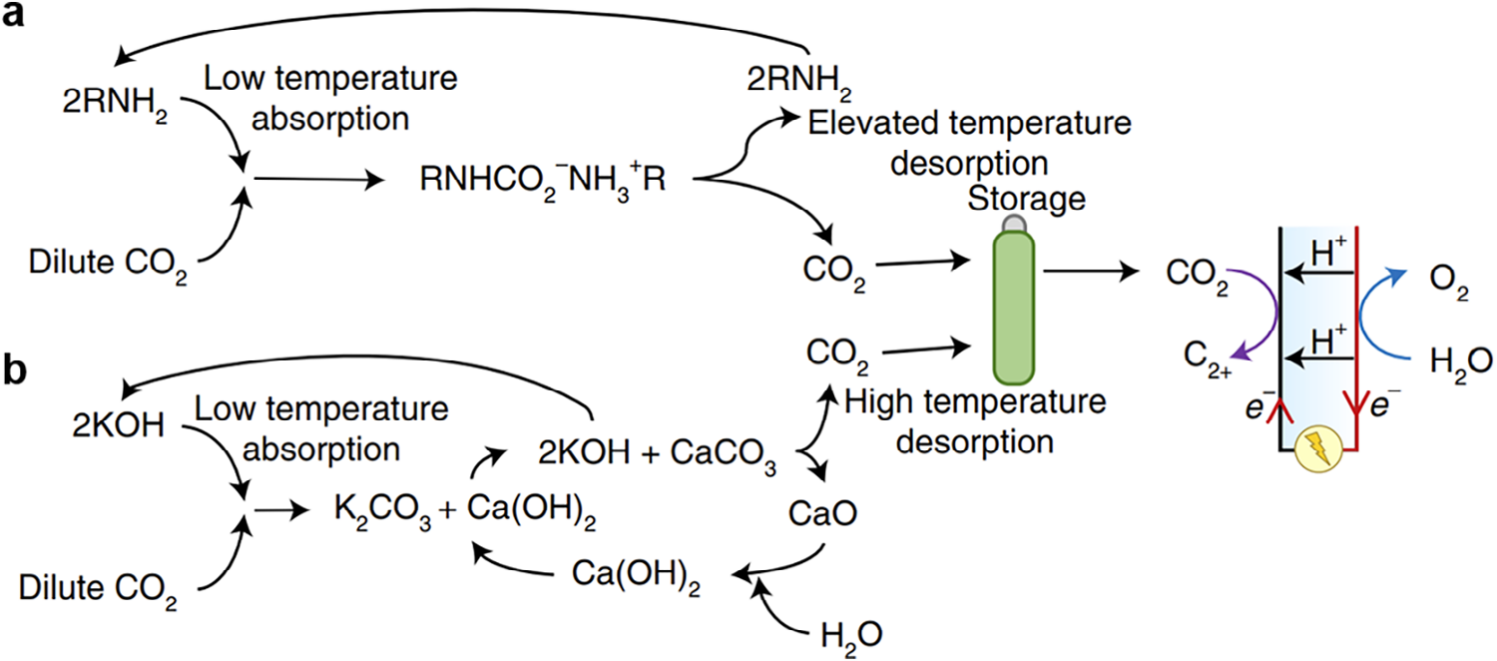
Type‐I, independent CO_2_RR and CO_2_ capture processes. Type‐I‐a, CO_2_ is captured by an amine‐based solution at low temperature, followed by amine regeneration and CO_2_ release via temperature swings. In Type‐I‐b, CO_2_ is captured by an alkaline solution to form carbonate species and is then converted into CaCO_3_ through the reaction with Ca(OH_2_). A thermochemical process then releases CO_2_ in a pure feed, which allows for use in CO_2_RR. Reproduced with permission.^[^ ^128^ ^]^ Copyright 2021, Springer Nature.

**Figure 8:**
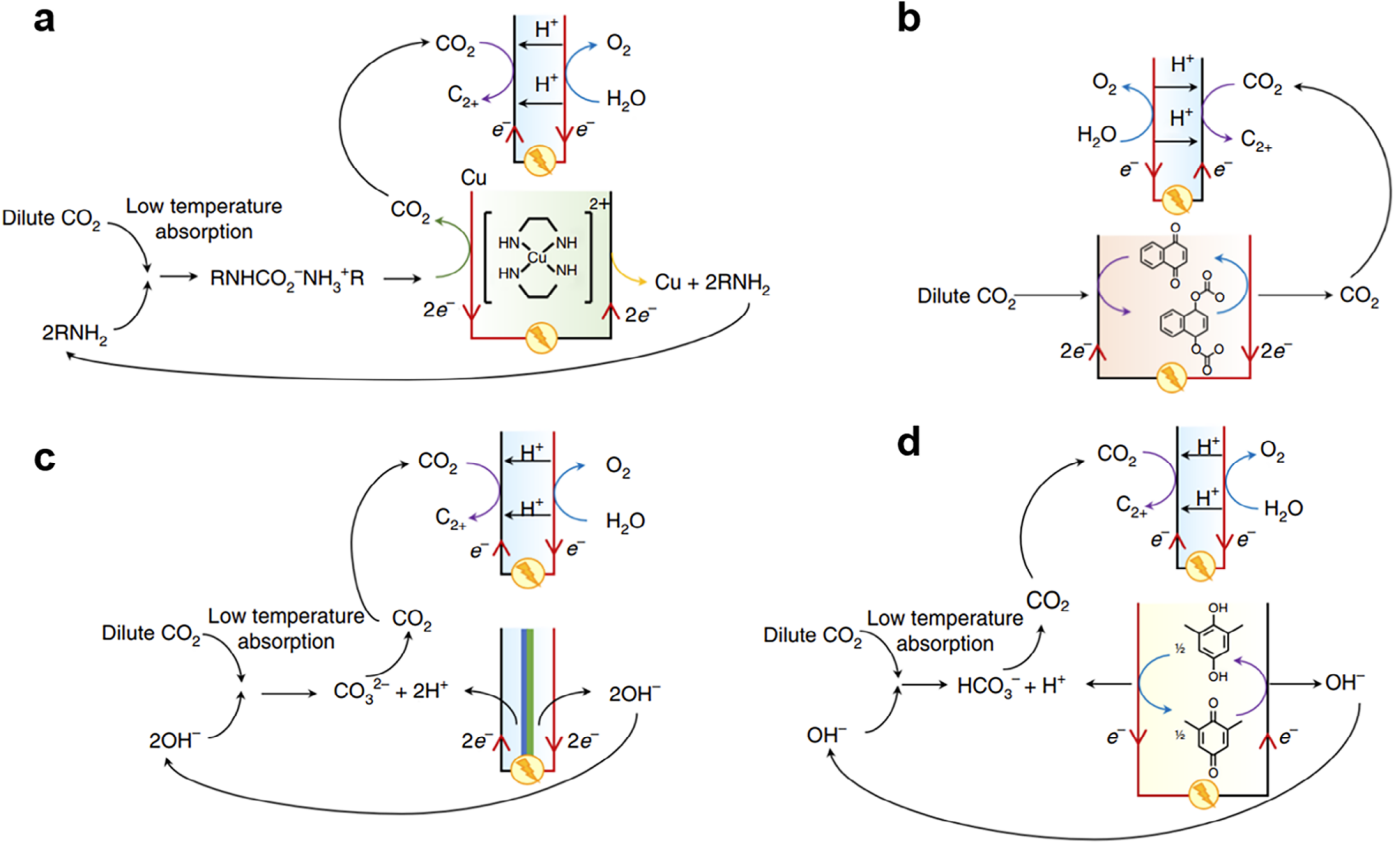
Type‐II, subsequent stage CO_2_RR and CO_2_ capture processes. Technologies for local coupling include electrochemically mediated amine regeneration (Type‐II‐a), redox active carrier (Type‐II‐b), BPM‐based electrodialysis (Type‐II‐c) and PCET‐based CO_2_ capture (Type‐II‐d). Type‐II‐a and b rely on anodic release of CO_2_ through a redox mediator, whereas Type‐II‐c and d rely on the local generation and reaction of protons to release CO_2_. Reproduced with permission.^[^ ^128^ ^]^ Copyright 2021, Springer Nature.

**Figure 9:**
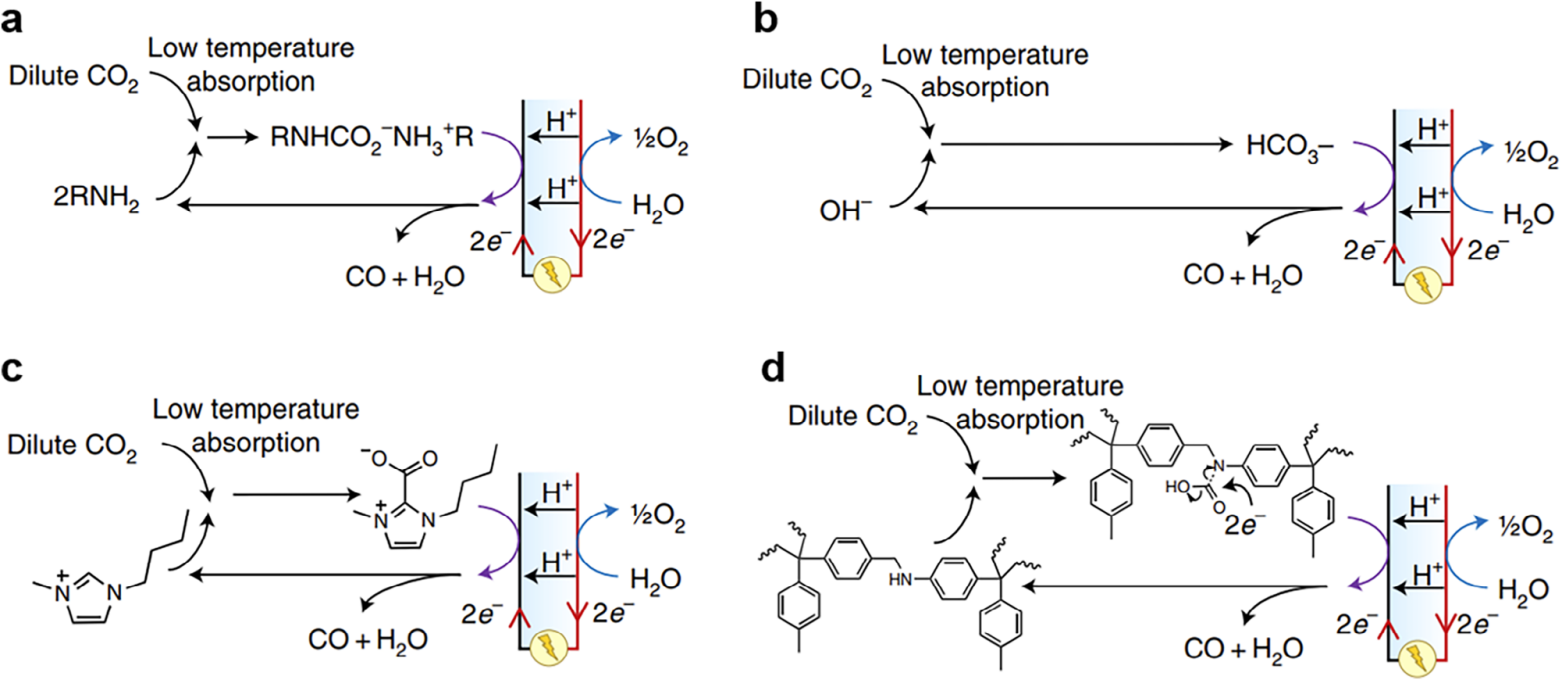
Type‐III, fully integrated CO_2_RR and CO_2_ capture processes. Direct electroreduction of CO_2_‐loaded capture agents based on amines (Type‐III‐a), bicarbonates and/or carbonates (Type‐III‐b), ILs (Type‐III‐c), and COFs (Type‐III‐d). Type‐III processes eliminate the capture media regeneration and molecular CO_2_ release step, which has the potential to improve the energy efficiency of the system and to lower the cost of the reduced products. Reproduced with permission.^[^ ^128^ ^]^ Copyright 2021, Springer Nature.

CO_2_RR can be coupled with CO_2_ capture through three levels of integration:^[^ ^128^ ^]^ Type‐I (fully separate), Type‐II (partially coupled), and Type‐III (fully integrated). In Type‐I, capture and conversion occur independently, such as Type‐I‐a using thermal amine regeneration and Type‐I‐b using alkaline regeneration (**Figure** **7**). Type‐II configurations release molecular CO_2_ near the conversion site and include methods such as Type‐II‐a (electrochemical amine regeneration), Type‐II‐b (redox‐active carriers), Type‐II‐c (bipolar membrane electrodialysis), and Type‐II‐d (PCET‐based capture) (**Figure** **8**). Type‐III systems bypass CO_2_ release entirely by directly reducing captured species, such as in Type‐III‐a (amines), Type‐III‐b (bicarbonates/carbonates), Type‐III‐c (ionic liquids), and Type‐III‐d (COFs) (**Figure** **9**). Type‐III benefits from lower overpotentials due to activated CO_2_ adducts but faces challenges like solvent degradation in oxygen‐rich streams.

**Figure 10:**
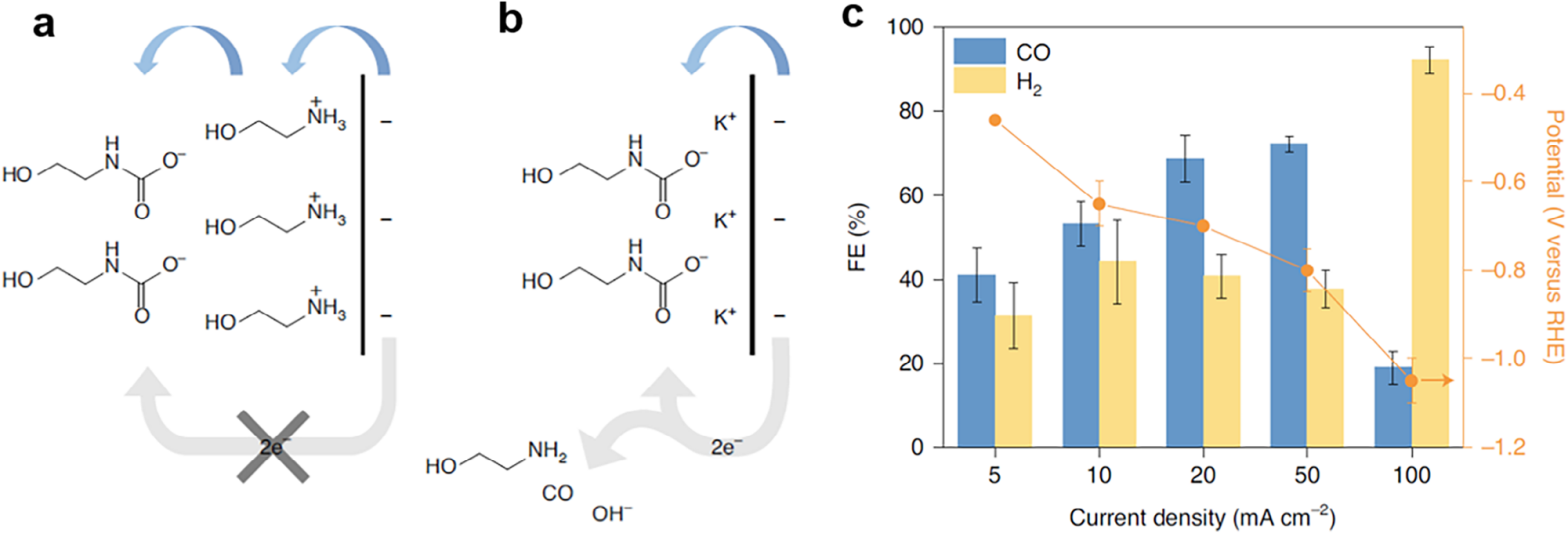
Proposed interfacial structure near the electrode surface. a) MEA–CO_2_ electrolyte. b) MEA–CO_2_ with alkali salt electrolyte. c) Product distribution of MEA–CO_2_ conversion to H_2_ and CO at different applied current densities, ranging from 5  to 100 mA cm^−2^ in a flow cell system. The error bars represent the standard deviation of three independent measurements. Reproduced with permission.^[^ ^129^ ^]^ Copyright 2021, Springer Nature.

Lee et al.^[^ ^129^ ^]^ proposed a novel electrochemical strategy that enables the direct reduction of CO_2_ from amine capture solutions, thus bypassing the desorption step entirely. Results show that use of the K^+^ ion leads to a more compact double layer on the electrode surface compared to the case of ethanolammonium cation (**Figure** **10**a,b), in agreement with the view that the molecular size of the ethanolammonium cation is larger than that of the hydrated K^+^ in the electrochemical double layer (EDL). In their system, CO_2_ is captured in a 30 wt% aqueous MEA solution and then directly electrochemically reduced to CO using a Ag catalyst. The key obstacle they overcame was the limited electrochemical activity of the bound CO_2_ species (mainly carbamates), which are not as easily reduced as gaseous CO_2_ and tend to remain too far from the electrode surface due to double‐layer effects. Their approach centered on tuning the electrochemical double layer at the cathode–electrolyte interface. By introducing alkali metal cations, particularly potassium ions (K⁺), they were able to compress the double layer, effectively bringing the carbamate species closer to the catalytic Ag surface and facilitating their electron transfer. This modification significantly enhanced the electrochemical conversion of captured CO_2_. Under optimized conditions, the system achieved a CO FE of 72% at a current density of 50 mA cm^−2^, representing one of the highest reported performances for direct CO_2_RR from capture media (Figure 10c).

## 6 CO_2_RR from Impurity‐Containing Flue Gas Streams

Industrial adoption of CO_2_RR technology requires rendering catalysts and systems tolerant to detrimental flue‐gas impurities, such as O_2_, NO_x_, and SO_2_. The field has thus focused on developing catalyst‐ and system‐level strategies to improve the impurity tolerance of CO_2_RR systems. The following subsections discuss the effects of common flue‐gas impurities on CO_2_RR performance and provide an overview of innovative catalyst and system design strategies with improved tolerance to flue‐gas impurities.

### 6.1 CO_2_RR from O_2_‐Containing Flue Gas Streams

Flue gas streams of many industrial processes contain notable O_2_ concentrations: 3–4% in the coal‐fired power plant, 2%–14% in that of cement production, 12–15% in that of natural gas combined cycle, and <1% in that of steel manufacturing (Table 1). Thus, assessing the influence of O_2_ impurities in CO_2_RR performance and stability, and developing effective mitigation strategies is required to enhance the industrial relevance of CO_2_RR systems. This section will provide an overview of emerging catalyst‐ and system‐design strategies for O_2_‐tolerant CO_2_RR.

#### 6.1.1 Selective Separation Strategy

Rational design of new materials and reactors with high CO_2_RR selectivity warrants a detailed physicochemical understanding of reaction mechanisms. A key strategy in this regard is optimizing mass transport by enhancing CO_2_ delivery while limiting O_2_ diffusion, thereby promoting CO_2_RR. Selecting an appropriate feedstock is a viable approach to blocking O_2_ transport and maintaining CO_2_ delivery.

**Figure 11:**
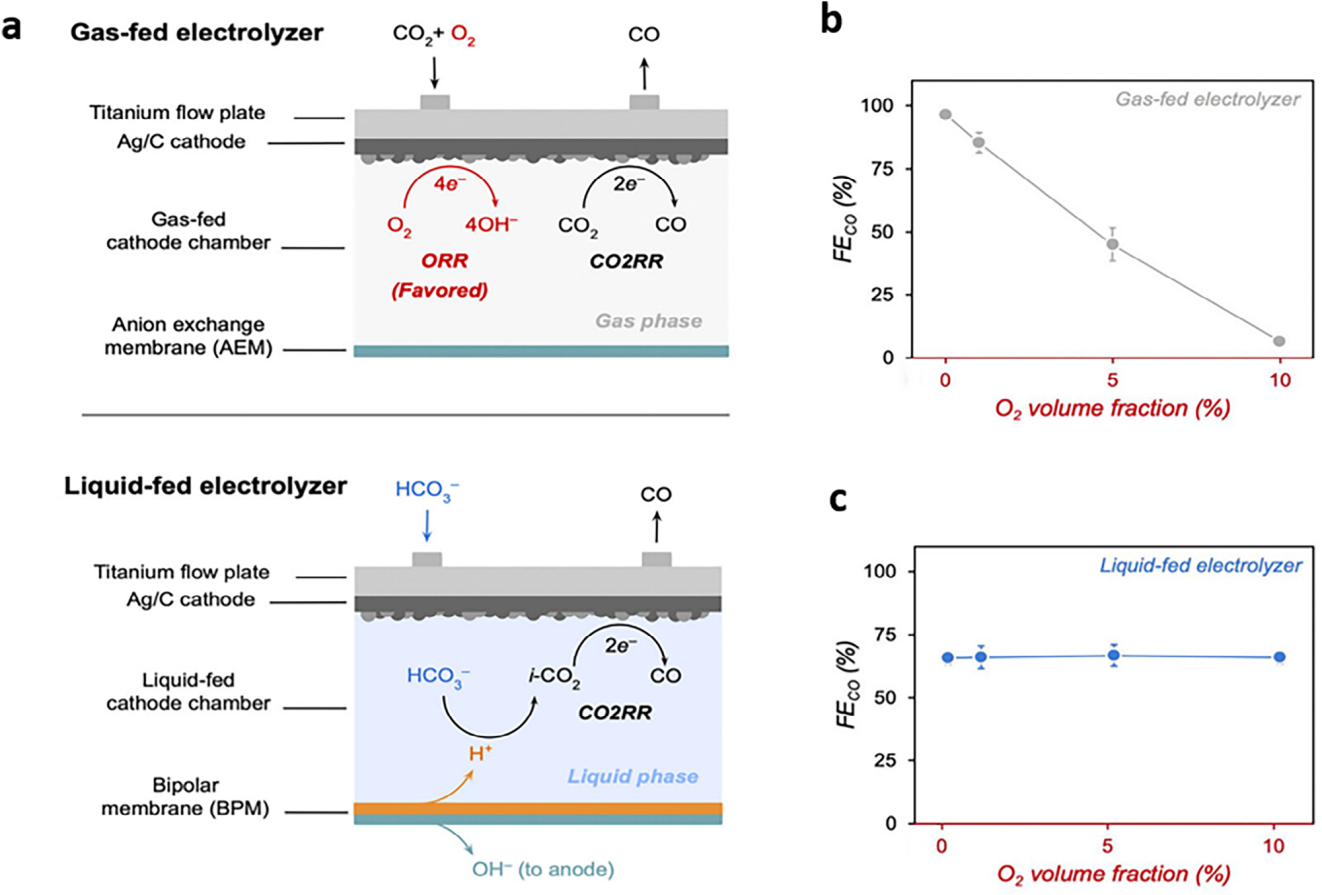
CO_2_RR from O_2_‐containing streams in gas‐fed and liquid‐fed electrolyzers. a) Schematic illustration of CO_2_RR in a gas‐fed CO_2_ electrolyzer (top) and a liquid‐fed bicarbonate electrolyzer (bottom). b) CO FEs following 5 min of CO_2_RR using a humidified CO_2_ stream containing 0–10% O_2_ at 100 mA cm^−2^. c) CO FEs following 5 min of CO_2_RR at 100 mA cm^−2^ using 3 m KHCO_3_ bubbled with CO_2_ containing 0%–10% O_2_. Reproduced with permission.^[^ ^24^ ^]^ Copyright 2023, ACS Publications.

Pimlott et al.^[^ ^24^ ^]^ demonstrated that liquid bicarbonate feedstocks enhance CO_2_RR by delivering high CO_2_ concentrations to the cathode while minimizing O_2_ interference through limiting its solubility in aqueous media (**Figure** **11**). This fosters a cathodic environment that preferentially facilitates CO_2_RR over ORR without additional O_2_ removal steps (Figure 11a). The mechanistic findings herein are validated through CO_2_RR to CO, achieving a CO FE of 65% at 100 mA cm^−2^ using 3 m KHCO_3_ solution bubbled with either 100% CO_2_ or 100% O_2_ (Figure 11c). In contrast, experiments with gaseous CO_2_ reveal that the presence of even 0.5% O_2_ in the feedstock causes a 90% reduction in CO FE during the initial 1 h of electrolysis (Figure 11b).

**Figure 12:**
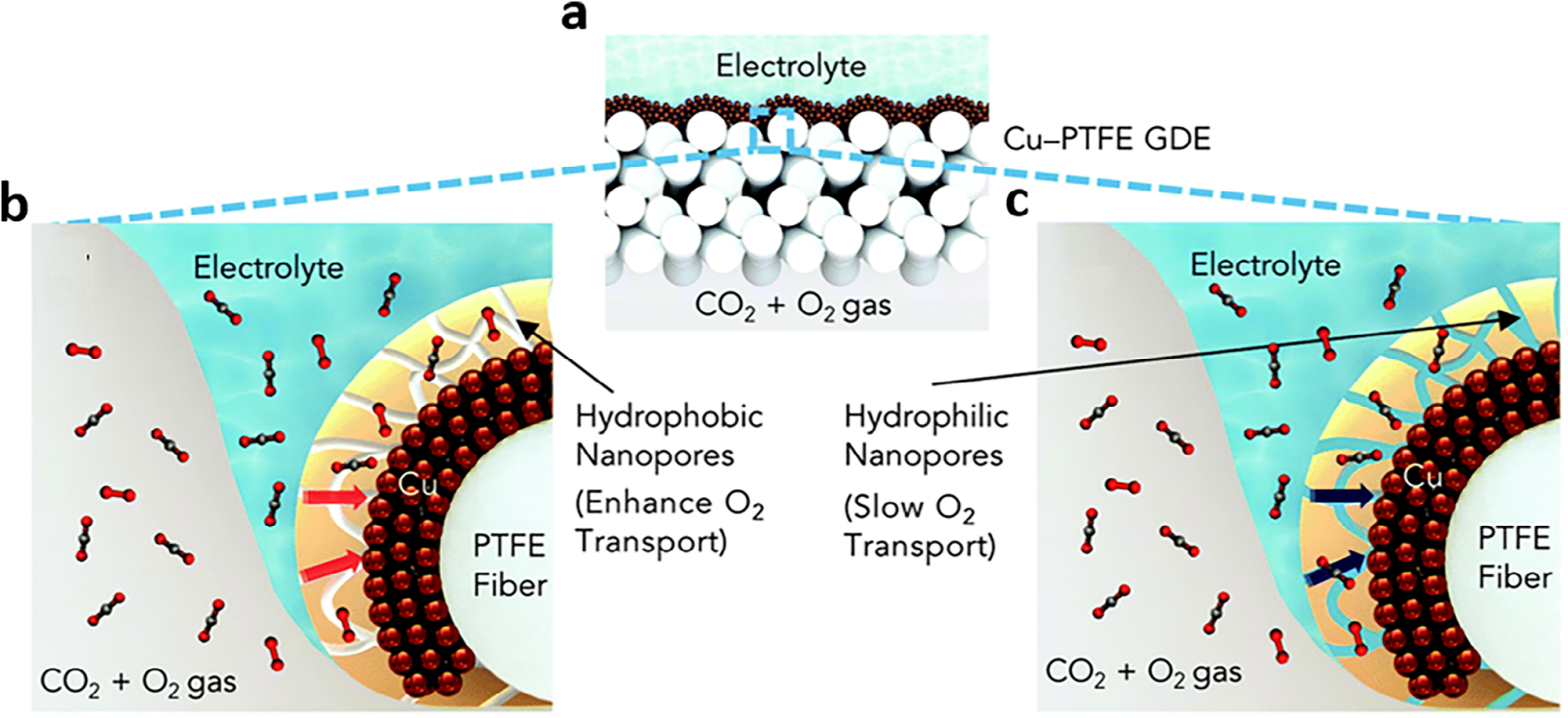
Effect of ionomer layer on the performance of CO_2_RR from pressurized (10 bar) streams containing 15% CO_2_ (v/v) and 4% O_2_ (v/v), 1 m KOH electrolyte, and TiO_2_ support particles. a) Schematic illustration of the Cu‐PTFE GDE. b) Schematic illustration of the micro‐reaction environment of the GDE coated with the hydrophobic nanoporous ionomer. c) Schematic illustration of the GDE coated with the hydrophilic nanoporous ionomer. Reproduced with permission.^[^ ^9^ ^]^ Copyright 2020, Royal Society of Chemistry.

Besides enhancing CO_2_RR, the functional groups of the decorative electrocatalyst can enrich local CO_2_ concentration. Xu et al.^[^ ^9^ ^]^ investigated the impact of O_2_ impurities on CO_2_RR performance using a Cu catalyst at high pressures. It is found that increasing pressure from 1 to 15 bar enhances CO_2_RR FE in 15% CO_2_ without O_2_; however, introducing 4% O_2_ at 15 bar dominates the ORR over CO_2_RR. The lower CO_2_RR FEs are ascribed to the increasing partial pressure of O_2_ and limited concentration of CO_2_ at active sites. The authors mitigated this challenge by modifying the surface of the Cu catalyst with a hydrated ionomer coating (**Figure** **12**a,b), where hydrophobic nanopores facilitate CO_2_ diffusion, while hydrophilic nanopores constrain O_2_ transport to the active sites without affecting CO_2_ permeability (Figure 12c). Consequently, the Cu catalysts modified with hydrophilic ionomers (i.e., Sustainion or Fumion) demonstrate higher CO_2_RR FEs compared to those modified with hydrophobic ionomers (i.e., Nafion or Aquivion), achieving a C_2+_ FE of 68% at 3 V.

**Figure 13:**
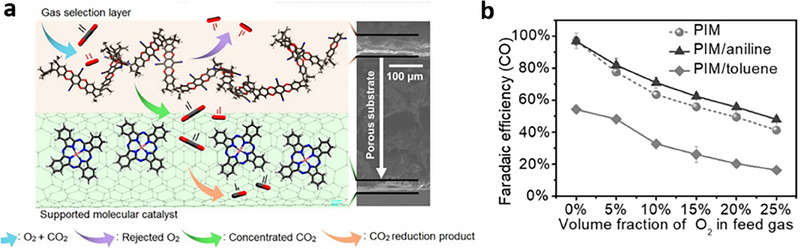
Promoting CO_2_RR from O_2_‐containing CO_2_ feeds via polymer modifications. a) Schematic diagram and cross‐sectional scanning electron microscopy (SEM) image of the architecture of the PIM‐CoPc/CNT hybrid electrode for O_2_‐tolerant CO_2_RR. The polymeric film augments the availability of CO_2_ molecules at active sites while constraining that of O_2_ molecules, promoting CO_2_RR. Atoms: black – carbon, red – O_2_, pink – cobalt, blue – N_2_. Reproduced with permission.^[^ ^26^ ^]^ Copyright 2019, Elsevier. b) CO FE for PIM, PIM/aniline, and PIM/toluene cathodes operating with CO_2_/O_2_ feed gas containing O_2_ with various concentrations. Reproduced with permission.^[^ ^23^ ^]^ Copyright 2019, Wiley‐VCH.

Polymeric materials could achieve selective CO_2_ adsorption owing to the functional groups in their structure or the structure's intrinsic microporosity. For instance, a polymer with favorable intrinsic microporosity could serve as a CO_2_‐selective layer, enabling a CO_2_/O_2_ selectivity of ≈20. Lu et al.^[^ ^136^ ^]^ developed an electrode containing a polymer film of intrinsic microporosity (PIM). The polymer layer possesses size‐selective pores capable of rejecting O_2_ molecules and permitting the passage of CO_2_ molecules (**Figure** **13**a). Integrated into a flow electrolytic cell, the hybrid electrode achieves a CO FE of 75.9% at a current density of 27.3  mA cm^−2^ and a cell voltage of 3.1 V using a CO_2_ stream containing 5% O_2_. The authors also discovered that incorporating aniline molecules into the pore structure of a polymer of intrinsic microporosity extends its gas separation functionality beyond pure physical sieving.^[^ ^23^ ^]^ The chemical interaction between the acidic CO_2_ molecules and the basic amino group of aniline enhances CO_2_ separation from O_2_. When combined with a cobalt phthalocyanine‐based catalyst, the hybrid electrode achieves a CO FE of 71% from a CO_2_ feed containing 5% O_2_ (Figure 13b).^[^ ^23^ ^]^ The polymer modification strategies—by mitigating the interference of O_2_ via physical adsorption—enhance CO_2_RR performance from O_2_‐containing streams. However, the surface modification using polymeric films presents several challenges. These include higher cell voltages, progressive blockage of active sites by the additional polymeric layers, stability loss due to ionomer swelling, operational complexities, and scalability challenges.

#### 6.1.2 Chemical Suppression of ORR

**Figure 14:**
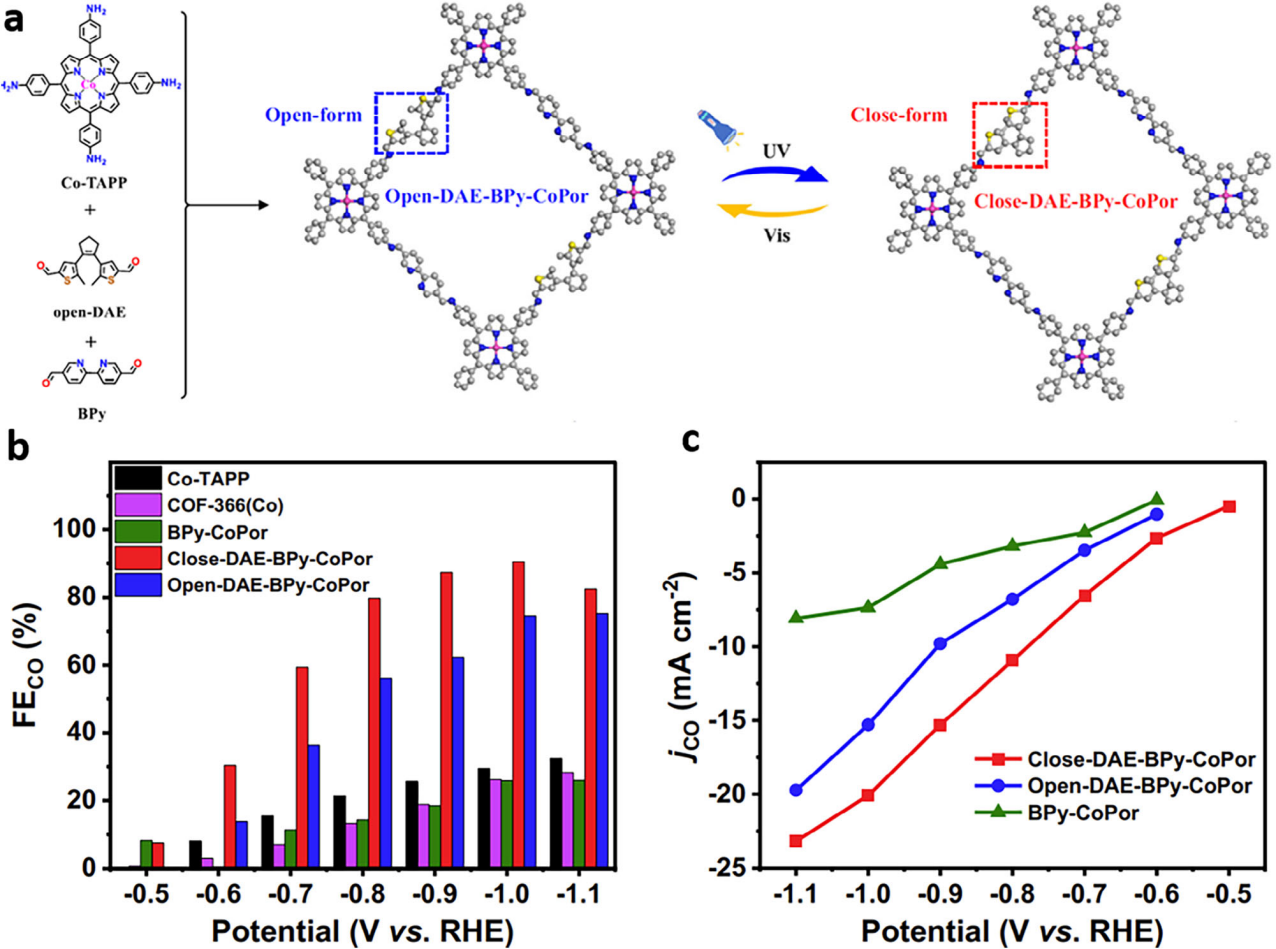
Promoting CO_2_RR via chemical suppression of ORR (O_2_ passivation strategy). a) Synthesis of open‐DAE‐BPy‐CoPor and close‐DAE‐BPy‐CoPor. b) CO FE on various catalysts performing CO_2_RR from O_2_‐containing CO_2_ feeds. c) *j*_CO_ of various catalysts in a wide range of applied potentials from O_2_‐containing CO_2_ feeds. Reproduced with permission.^[^ ^25^ ^]^ Copyright 2024, Springer Nature.

Chemical suppression of ORR—compared to physical adsorption—presents a more effective strategy, providing a significant advantage for CO_2_RR from O_2_‐containing CO_2_ feeds. Recently, Zhu et al.^[^ ^25^ ^]^ introduced an O_2_ passivation strategy utilizing a diarylethene (DAE) molecule. The DAE molecule suppresses the electron transfer to O_2_ in its ring‐closed form, and it undergoes reversible conversion between its open (visible light) and closed (UV light) forms. This reversible transformation causes energy transfer variations between O_2_ and active sites (**Figure** **14**a). From a CO_2_ feed containing 5% O_2_, a cobalt porphyrin‐DAE catalyst achieves a 90.5% CO FE with a *j*_CO_ of 20.1 mA cm^−2^, outperforming the 25.9% CO FE observed in the absence of DAE (Figure 14b,c).

#### 6.1.3 Suppression of ORR via pH Effects

**Figure 15:**
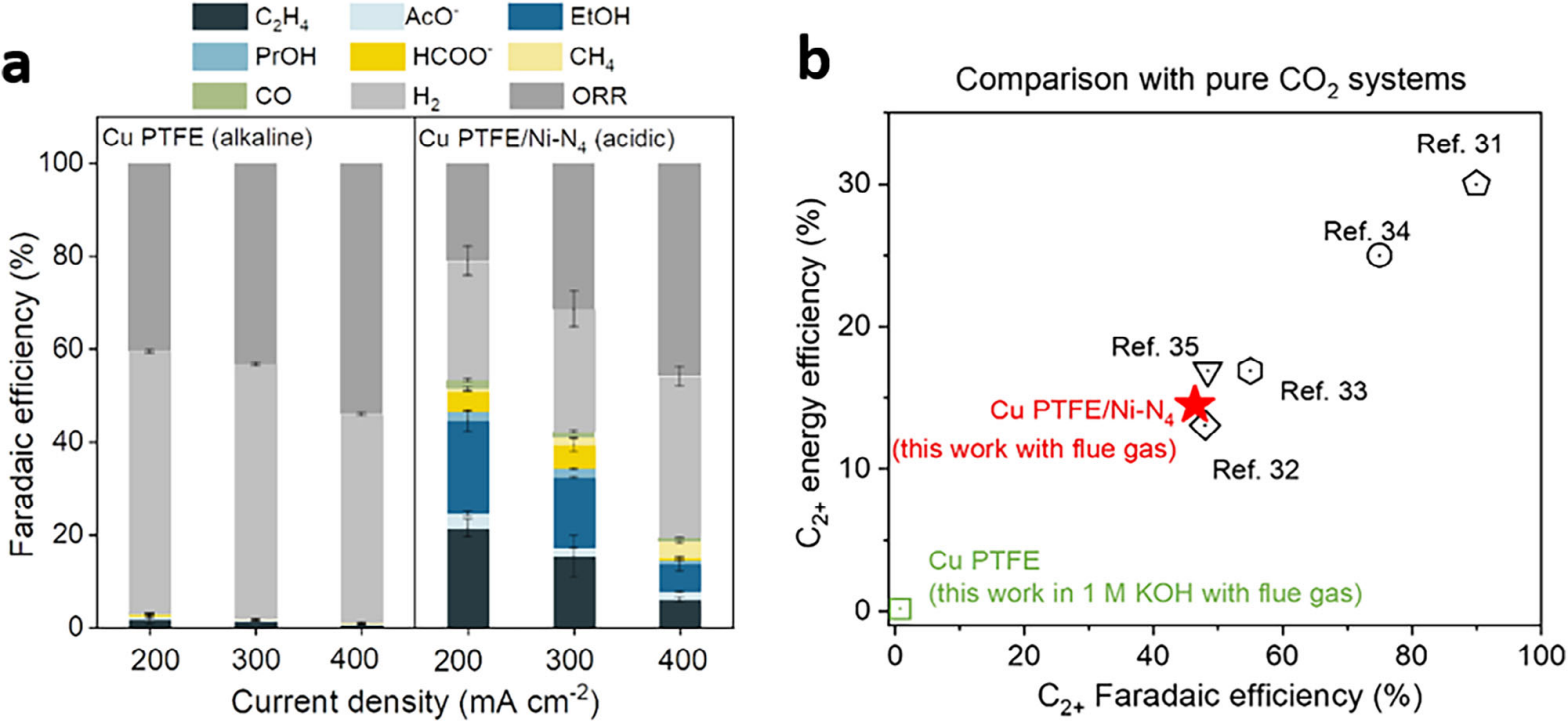
Promoting O_2_‐tolerance of CO_2_RR systems using acidic electrolytes. a) CO_2_RR products on various CO_2_RR catalysts. Cu PTFE in 1 m KOH and Cu PTFE/Ni–N_4_ in 0.05 m H_2_SO_4_ + 1.5 m Cs_2_SO_4_ at various current densities. b) Comparison of the C_2+_ EE and C_2+_ FE of the O_2_‐tolerant acidic‐media CO_2_RR system with those of literature benchmark systems. Reproduced with permission.^[^ ^27^ ^]^ Copyright 2024, Springer Nature.

The specifications and pH of the electrolyte could modulate CO_2_RR kinetics from O_2_‐containing CO_2_ streams. Wang et al.^[^ ^27^ ^]^ demonstrated that an acidic electrolyte can effectively suppress the ORR on a Cu catalyst, promoting C_2+_ products from simulated flue gas. The use of acidic electrolytes and a composite catalyst comprised of Cu and carbon‐supported Ni SAC in a tandem configuration enables a C_2+_ FE of 46.5% at 200 mA cm^−2^. This performance represents an approximately 20‐fold enhancement compared to that of bare Cu under alkaline conditions (**Figure** **15**a). Notably, the acidic‐media, flue‐gas‐fed system shows performance comparable to that of acidic‐media pure‐CO_2_‐fed CO_2_RR systems (Figure 15b).

### 6.2 CO_2_RR from NO_x_‐Containing Flue Gas Streams

**Figure 16:**
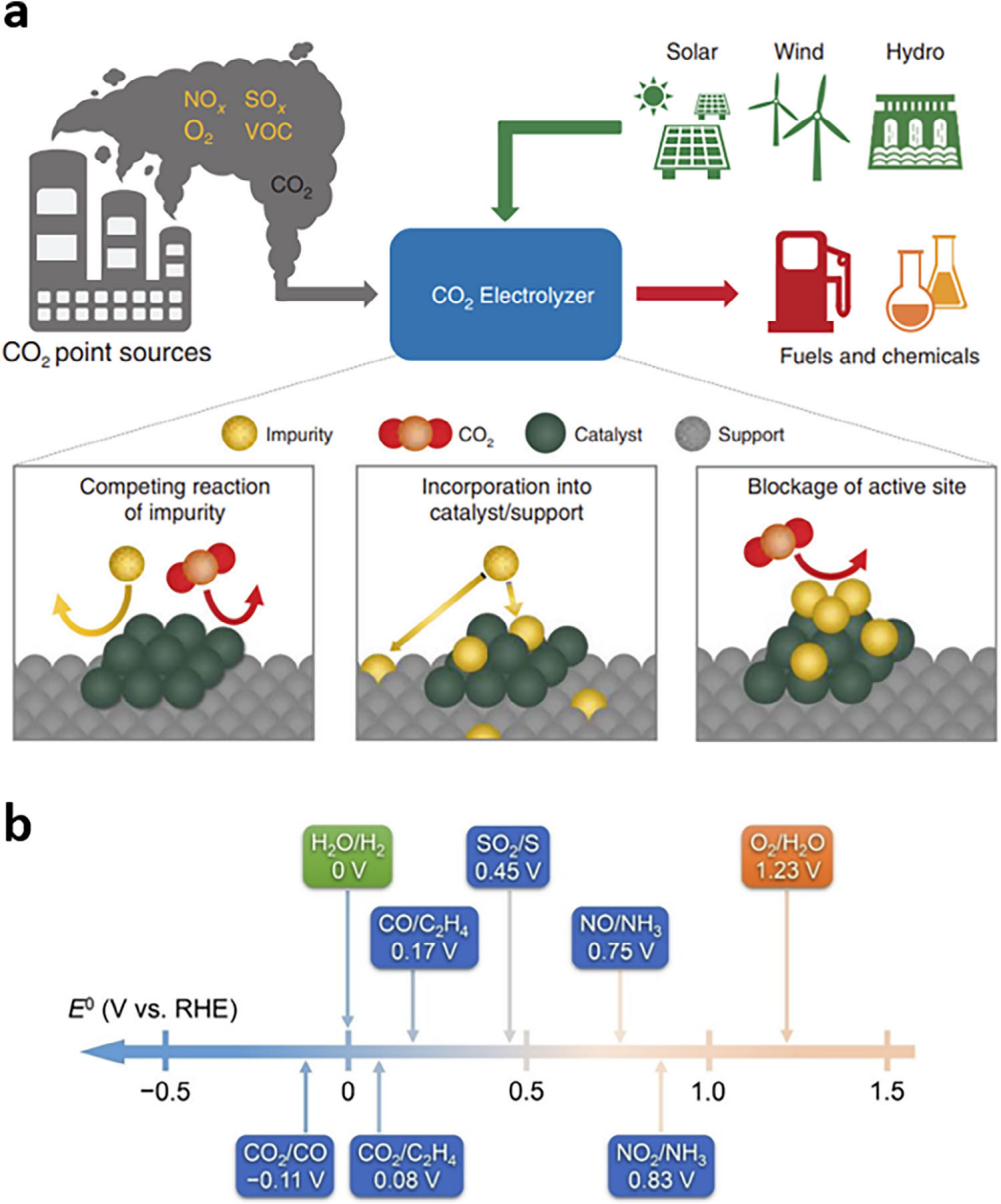
Renewable‐electricity‐powered CO_2_RR to fuels and chemicals using point CO_2_ sources. a) Schematic illustration of CO_2_RR from point sources containing common flue‐gas impurities, such as SO_x_, NO_x_, and O_2_. Reproduced with permission.^[^ ^45^ ^]^ Copyright 2020, Springer Nature. b) Standard electrode overpotentials vs RHE for CO_2_RR, HER, SO_2_RR, and NO_2_RR. Reproduced with permission.^[^ ^31^ ^]^ Copyright 2025, Wiley‐VCH.

NO_x_ is a flue gas impurity that exists in various streams (**Figure** **16**a). NO_x_ is comprised of 90%–95% NO and 5%–10% NO_2_,^[^ ^31^ ^]^ and typically exists at concentrations of 1000 ppm.^[^ ^45^ ^]^ Early works on NO_x_ impurities focused on investigating the concentration‐dependent impact of NO_x_ impurities on CO_2_RR performance. Zhai et al.^[^ ^137^ ^]^ explored the influence of NO_x_ impurities as a function of geometry, flow rate, and specification in solution. The presence of NO_2_ with concentrations of ≤1667 ppm either positively or negatively influences the CO_2_RR performance. However, increasing the concentration beyond 1667 ppm adversely influences CO_2_RR due to reduced electrolyte alkalinity. More recently, Ko et al.^[^ ^45^ ^]^ investigated the effect of NO_x_ impurities (i.e., NO, NO_2_, and N_2_O) on the CO_2_RR performance of various catalysts (i.e., Ag, Sn, and Cu) at practical current densities of >100 mA cm^−2^ in a three‐electrode flow cell. The presence of NO_x_ impurities in the CO_2_ stream suppresses CO_2_RR. NO_x_ impurities possess a much more positive electrochemical reduction potential than CO_2_ (Figure 16b), and their reduction initiates before CO_2_RR. From a performance standpoint, NO and NO_2_ impurities are more deteriorative than N_2_O, attributable to the need for a greater number of electrons in the NO_x_ reactions. The electroreduction of NO_x_ species enables the production of nitrous oxide, nitrogen, hydroxylamine, and ammonia. During electrolysis, the presence of NO_x_ species negatively impacts the CO_2_RR. Upon the removal of NO_x_ species from the stream, the CO_2_RR FE recovers substantially, approaching to the CO_2_RR FE obtained from a pure CO_2_ stream. This observation implies that the loss in CO_2_RR performance—induced by the NO_x_ species—is largely reversible.

### 6.3 CO_2_RR from SO_2_‐Containing Flue Gas Streams

Flue gas streams of many industrial processes contain ppm‐level SO_2_: 10–1800 ppm in the stream of coal‐fired power plant, 100–1300 ppm in that of cement production, <10 ppm in that of natural gas combined cycle, and trace amounts in that of steel manufacturing (Table 1). SO_2_ impurities can be removed from streams via gas scrubbing. However, even after gas scrubbing, up to 10 ppm of SO_2_ typically remains in the streams.^[^ ^138^ ^]^ Even a trace amount of SO_2_ could substantially reduce the performance, stability, and applicability of direct flue gas utilization in CO_2_RR systems.

Luc et al.^[^ ^139^ ^]^ explored the impact of SO_2_ on Ag, Sn, and Cu‐based catalysts in neutral‐media flow cells. Results show that SO_2_ decreases CO_2_RR FE, due to the preferential reduction of SO_2_. The long‐term effect of SO_2_ impurities depends on the catalyst type. Although the poisoning effect is recoverable on Ag and Sn catalysts, it is irreversible on Cu catalysts, revealing the low tolerance of Cu catalysts to SO_2_. On Ag and Sn catalysts, the presence of 1% SO_2_ in the CO_2_ stream decreases the CO_2_RR FE, suggesting that a portion of the electrons is used in the reduction of SO_2_ in lieu of CO_2_RR. From the thermodynamics standpoint, the CO_2_RR is less favorable than SO_2_ reduction on Ag and Sn catalysts:

(5) $$SO_{2}+4H^{+}+4e^{-}\to S+2H_{2}O,\;E^{o}=0.50\;V$$

(6) $$CO_{2}+2H^{+}+2e^{-}\to CO+H_{2}O,\;E^{o}=-0.11\;V$$

(7) $$CO_{2}+2H^{+}+2e^{-}\to HCOOH,\;E^{o}=-0.25\;V$$

Co‐utilization of computational tools and X‐ray photoelectron spectroscopy (XPS) reveals that the formation of Cu_2_S causes poisoning of the Cu surface, shifting the selectivity from C_2+_ products to formate. These results indicate that CO_2_ from DAC or biorefineries—in the light of their SO_2_‐free natures—could be more suitable for electrosynthesis of C_2+_ products on Cu catalysts.

**Figure 17:**
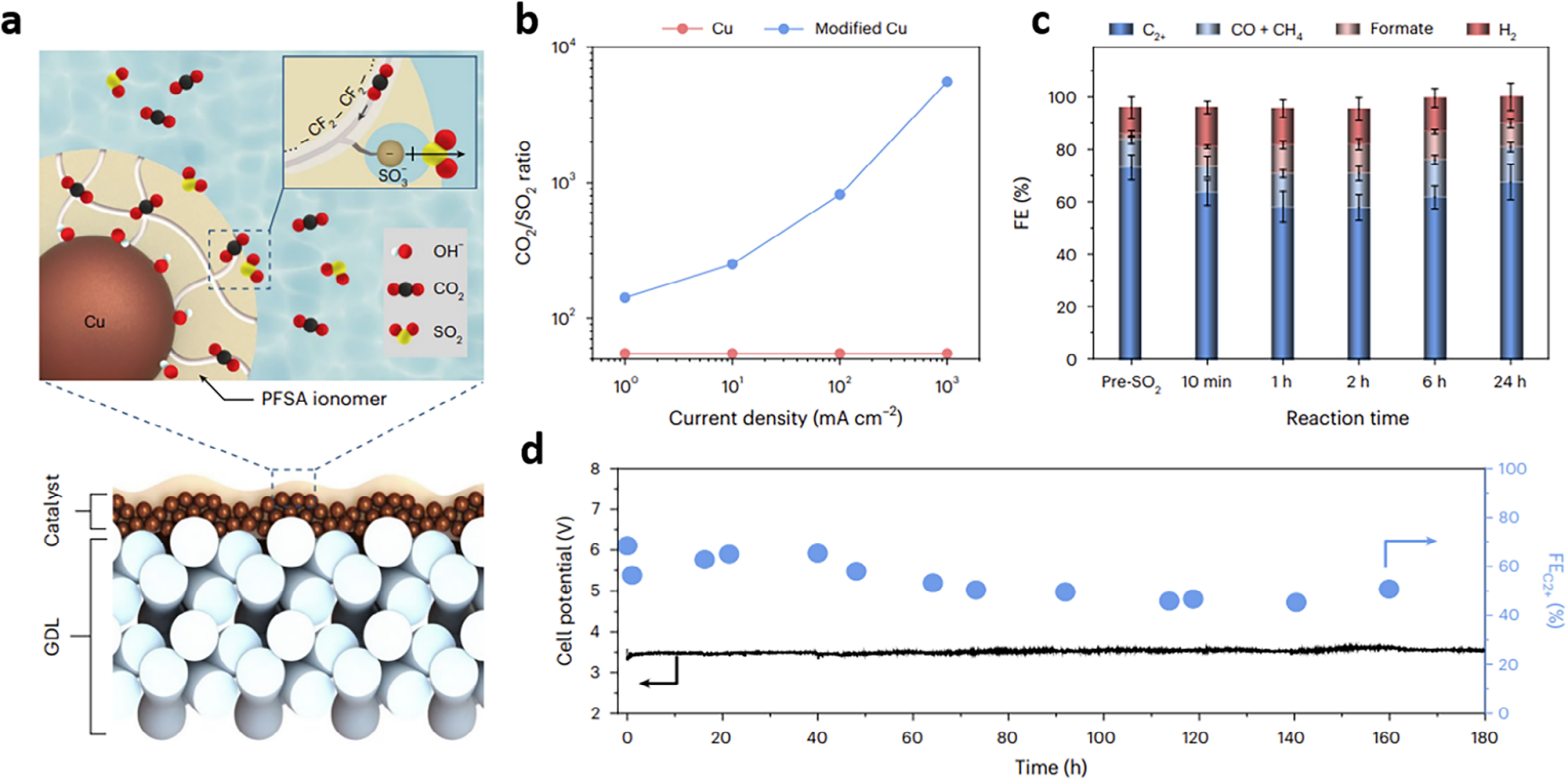
Architecture and catalytic performance of a catalyst for SO_2_‐tolerant CO_2_RR. a) Schematic illustration of a catalyst/polymer/ionomer heterojunction. The top panel illustrates the micro‐reaction environment of the modified Cu catalyst. b) Model showing the effect of ionomer modification on the mass flux ratios of CO_2_ over SO_2_ at different reaction rates during CO_2_RR from SO_2_‐containing CO_2_ streams. c) Time‐dependent FE distribution of modified Cu catalyst. d) Extended CO_2_RR from SO_2_‐containing (400 ppm SO_2_) dilute CO_2_ streams (50%, balanced with N_2_). Reproduced with permission.^[^ ^28^ ^]^ Copyright 2024, Springer Nature.

Papangelakis et al.^[^ ^28^ ^]^ sought to uncover the roots of low C_2+_ FE in the co‐electrolysis of CO_2_/SO_2_. Computational studies highlight that SO_2_ hydrogenates with adsorbed hydrogen (*H), causing the accumulation of *H and *S species on the Cu catalyst. This accumulation blocks the active sites, hindering CO_2_ adsorption and dominating competing HER. In turn, this causes a decline in C_2+_ FE, typically shifting CO_2_RR toward formate. Computational studies also ascribe the increasing formate FE to the reaction of CO_2_ with *H instead of solvated H. This, in turn, causes a decrease in CO formation and its ensuing dimerization—a critical step toward C_2+_ products. Meanwhile, SO_2_ reduction causes simultaneous accumulation of *H and *S species on the Cu surface, reducing C_2+_ FE. Encouraged by these mechanistic insights, the authors developed a heterojunction consisting of a catalyst/polymer/ionomer that integrates hydrophobic and charged hydrophilic structures to address SO_2_ poisoning (**Figure** **17**a). The catalyst design controls hydrogen adsorption, promoting CO_2_ transport over the SO_2_ transport. Modeling studies estimate a 10‐fold increase in the CO_2_/SO_2_ mass flux ratio for the modified Cu catalyst (Figure 17b). Accordingly, the modified Cu catalyst sustains a 60% C_2+_ FE at a constant current density of 100 mA cm^−2^ for over 24 h from a CO_2_ stream containing 400 ppm SO_2_ (Figure 17c). Further, the modified Cu catalyst achieves 160 h of CO_2_RR with a sustainable C_2+_ FE of 50% from a CO_2_ stream containing 400 ppm SO_2_. Throughout extended CO_2_RR, the modified Cu catalyst maintains an *iR*‐free full‐cell potential of 3.5 V at 100 mA cm^−2^ (Figure 17d).

Tian et al.^[^ ^39^ ^]^ sought to assess the influence of flue‐gas impurities from the viewpoints of dilution, competition, micro‐reaction environment tuning, and catalytic performance loss. The competition and dilution effects are identified as the performance‐deteriorating factors. The authors sought to mitigate these two factors through a catalyst design approach. The authors designed Ni‐based SACs that offer high atomic utilization, isolated active sites, and a tunable coordination environment. The Ni‐based SAC maintains a CO FE of 92% at current densities of 80–90 mA cm^−2^ from CO_2_ streams containing 0.6% SO_2_. Simulation findings reveal that acidic impurities could improve local CO_2_ concentration by mitigating carbonate formation (through local pH modulation), elucidating the 18.8% enhancement in CO_2_RR FE. Experimental studies suggest the formation of sulfur–carbon (S–C) bonds on the catalyst surface, reflected as the U‐shaped trend in CO_2_RR FE with increasing SO_2_ composition. Results confirm the high‐speed cyclic scouring approach as a strategy to mitigate the performance‐degrading effects of SO_2_ poisoning.

## 7 Concluding Remarks

Given the low CO_2_ concentration in real flue gas (i.e., ≈15%), robust electrocatalysts that function efficiently at ambient conditions are essential for scalable CO_2_RR implementation. Recent breakthroughs in material science, reaction environment engineering, and electrolyte engineering have enhanced the impurity‐tolerance of CO_2_RR systems, improving their industrial adaptation. Such advances are mostly enabled by impurity‐tolerant catalyst and system/process design strategies across a range of system architectures:

- For direct utilization of dilute CO_2_ streams, emerging CO_2_RR systems require highly active and high‐surface‐area catalysts, preferably with high‐energy barriers for HER and with suitable micro‐reaction environments.
- For flue‐gas conversion via the BRR, multiple challenges remain. While innovative catalyst and system design approaches are critical to steering reaction toward target products at greater rates and energy efficiencies, other factors are limiting selectivity and energy efficiency. Major challenges in BRR include developing a more refined fundamental understanding, which can enable the development of new strategies for selectivity to be competitive with the analogous CO_2_RR. In terms of energetics and stability, the presence of the BPM currently represents a major limitation that raises cell voltages and potentially limits long‐term stability. Finally, the need to integrate the consumption of bicarbonate in the electrolyte with DAC warrants further consideration, potentially requiring an intermediate pH swing process.
- Impurity tolerance may require a suite of solutions. For O_2_‐tolerant CO_2_RR, emerging strategies involve O_2_ passivation, CO_2_ concentration enrichment, O_2_ exclusion via microporous polymers, and ORR suppression via acidic electrolytes. For SO_2_‐tolerant CO_2_RR, innovative catalyst design approaches involve the co‐utilization of ionomer and polymer films to promote CO_2_ and suppress SO_2_ transport.

Despite encouraging progress, the catalyst and system design innovations are still at the proof‐of‐concept stage, and the impact of flue‐gas impurities on product selectivity and catalyst stability remains elusive for many catalysts, systems and operating conditions. Further experimental, computational, and characterization efforts will be critical to identifying the impact of flue gas impurities on catalytic activity, stability, and catalyst degradation. Rationalizing these effects will motivate strategies that could effectively manipulate reaction environment and transport of CO_2_ and flue gas impurities to active sites without compromising voltage, energy efficiency, and stability. Additionally, systematic and parametric studies tracking and revealing structural changes and deactivation mechanisms will be critical to developing mitigation strategies. Further investigations via operando techniques, such as electrochemical transmission electron microscopy (EC‐TEM), X‐ray absorption spectroscopy (XAS), Raman spectroscopy, and infrared spectroscopy, will be further instrumental in gaining precise insights into the micro‐reaction environment in the co‐existence of CO_2_ and flue‐gas impurities. Parametric characterization studies will further elucidate the influence of flue‐gas impurities on various phenomena, including local CO_2_ concentration and availability, local pH and temperature, intermediate adsorption, surface coverage, and catalyst evolution. Additionally, operando characterization techniques should be utilized for parametric studies to reveal the effects of operating conditions on CO_2_RR activity and stability in the presence of O_2_. For example, understanding the activity and degradation patterns of catalysts for various impurity (NO_x_/SO_2_/O_2_) concentrations, operating temperatures, pressures, electrolyte specifications, and flow rates will guide further performance and stability advances. The dearth of computational and mechanistic studies presently limits our understanding of local reaction environments and variations in electrocatalytic properties in the presence of flue gas impurities. Therefore, operando characterization approaches should be further coupled with simulation and computational approaches to enrich mechanistic insights.

From a stability standpoint, the impurity‐tolerant CO_2_RR catalysts and systems have been mostly tested for limited durations, typically shorter than 160 h. Extending the CO_2_RR testing beyond 1000 hours will be insightful for understanding the effectiveness of impurity‐tolerance‐enhancing strategies for prolonged CO_2_RR. Such diagnostic studies will also be instrumental in understanding any stability‐limiting effect of today's benchmark impurity‐tolerant catalysts/systems during prolonged CO_2_RR. Additionally, the impurity‐tolerant catalyst design strategies should be further considered and improved from a scalability standpoint. The utilization of an additional ionomer layer for improved CO_2_ adsorption likely imposes scalability limitations by reducing through‐ and in‐plane electron transport throughout the GDE and introducing processing/manufacturing difficulties. Parallelizing experimental, computational, and characterization efforts will be critical to rendering CO_2_RR technology compatible with hard‐to‐abate flue gas impurities.

Lastly, each flue‐gas conversion technology utilizes distinct catalysts and systems, targeting various products. This, in turn, renders a substantial variance in performance metrics, including cell voltage, current density, and operational stability. The catalyst, system, and performance differences induce a substantial variance in techno‐economics, rendering it highly challenging for researchers to put forward fair comparative techno‐economic assessments. Excluding a few system‐specific techno‐economics studies, the literature presently lacks comprehensive techno‐economic analyses. Moving forward, each technology will benefit from further technological advances and component/system standardization. This will provide the datasets required for comparative techno‐economic assessments, which will be a critical milestone toward revealing systems, processes, and products with the highest techno‐economic potential in flue‐gas conversion.

## Conflict of Interest

The authors declare no conflict of interest.
